# Acquisition and Use of ‘Priors’ in Autism: Typical in Deciding Where to Look, Atypical in Deciding What Is There

**DOI:** 10.1007/s10803-020-04828-2

**Published:** 2020-12-29

**Authors:** Fredrik Allenmark, Zhuanghua Shi, Rasmus L. Pistorius, Laura A. Theisinger, Nikolaos Koutsouleris, Peter Falkai, Hermann J. Müller, Christine M. Falter-Wagner

**Affiliations:** 1 Department of Psychology, Ludwig-Maximilians-Universität München, Leopoldstr. 13, 80802 Munich, Germany; 2 Department of Psychiatry and Psychotherapy, Ludwig-Maximilians-Universität München, Munich, Germany

**Keywords:** Visual attention, Visual search, Predictive coding

## Abstract

Individuals with Autism Spectrum Disorder (ASD) are thought to under-rely on prior knowledge in perceptual decision-making. This study examined whether this applies to decisions of attention allocation, of relevance for ‘predictive-coding’ accounts of ASD. In a visual search task, a salient but task-irrelevant distractor appeared with higher probability in one display half. Individuals with ASD learned to avoid ‘attentional capture’ by distractors in the probable region as effectively as control participants—indicating typical priors for deploying attention. However, capture by a ‘surprising’ distractor at an unlikely location led to greatly slowed identification of a subsequent target at that location—indicating that individuals with ASD attempt to control surprise (unexpected attentional capture) by over-regulating parameters in post-selective decision-making.

**Supplementary Information:**

The online version contains supplementary material available at 10.1007/s10803-020-04828-2.

## Introduction

Individuals with ASD exhibit a range of sensory atypicalities, such as focus on details or superior performance on perceptual tasks (Dakin and Frith 2005; Leekam et al. 2007), making the issue of ‘How can sensory processing in autism be better understood?’ one of the top ten research priorities as assessed by individuals with Autism Spectrum Disorder (ASD) and stakeholders (Autistica 2016). The emergent idea is that these atypicalities reflect altered perceptual-inference processes, which may be constitutive of the autistic cognitive profile at large. While the ‘predictive-coding’ framework (Friston 2010; Friston and Kiebel 2009) offers a promising approach towards a principled understanding of these alterations, exactly which perceptual-decision processes are altered in ASD remains controversial (Brock 2012; Lawson et al. 2014; Pellicano and Burr 2012; Van de Cruys et al. 2014). Also, predictive coding in ASD has thus far only been examined for explicit decisions on stimuli that are in the focus of attention, that is, decisions at a post-selective stage of attentional processing, in terms of ‘classical’ architectures of visual selective attention (e.g., Koch and Ullman 1985; Treisman and Gelade 1980; Wolfe 2007). But little is known regarding more implicit decisions—at a pre-selective, or pre-attentive, stage—on which stimuli to bring into the focus of attention. This is surprising in view of recent attentional accounts of ASD (Keehn et al. 2013, 2016; Spaniol et al. 2018), according to which attentional abnormalities, in particular, a deficit in attentional reorienting (Orekhova and Stroganova 2014), influence developmental trajectories with far-reaching consequences for social-cognitive development. For instance, abnormal orienting of attention and over-reliance on sensory input rather than prior knowledge by individuals with ASD could impact their information processing during social interactions in developmentally important learning phases, resulting in cascading effects on social-cognitive functioning. Given this, the present study was designed to elucidate (1) which particular predictive-coding processes are altered in ASD and (2) whether the alterations already impact pre-selective decisions determining where to allocate focal attention, as well as post-selective decisions on the objects at the attended locations.

### Atypical Predictive Coding in ASD?

Predictive-coding accounts of perception (Friston 2010; Friston and Kiebel 2009) assume that the brain continually makes predictions, based on prior knowledge, about the environmental causes of the sensory inputs it receives. If a discrepancy between the top-down prediction and the actual sensory input (i.e., a prediction error) occurs, the brain attempts to reduce this mismatch by integrating the top-down prior information and the sensory input and adjusting its internal generative model accordingly. In this framework, prediction errors should be weighted by their precision. Errors arising from noisy, high-variance, sensory input would have low precision: as they do not provide reliable evidence that predictions can be improved by adjusting the internal model, they should receive only a low weight. In contrast, errors of high precision would be indicative of a systematic problem with predictions and should thus receive a high weight. The predictive-coding framework assumes that the precision is learnt from experience, and the weight assigned to prediction errors arising from any particular sensory source and context reflects the precision that an observer has learnt to associate with such inputs within this context.

While this general framework promises to yield a principled understanding of the perceptual atypicalities in ASD, opinions diverge with regard to how the differences between ASD and typically developed (TD) individuals arise. While the various accounts proposed agree that, in autism, perception is less influenced by prior knowledge, they disagree on the precise reasons why this is so. Pellicano and Burr’s (2012) ‘hypo-prior’ account assumes that perceptual atypicalities, and potentially many of the other atypicalities, arise because ASD individuals acquire broader, less specific, priors (termed ‘hypo-priors’); that is, their perception is less influenced by prior knowledge because their prior knowledge is less reliable. One alternative proposal is that sensory noise is reduced in ASD, compared to TD, individuals, thus making perceptual decisions less reliant on prior information (Brock 2012), but this has not been supported experimentally (Skewes et al. 2014; Skewes and Gebauer 2016). A second alternative assumes that individuals with ASD assign an unduly high weight to prediction errors, which is unwarranted given the level of uncertainty prevailing within a given context (Lawson et al. 2014; Van de Cruys et al. 2014). In line with this account (henceforth referred to as ‘precision-regulation’ account), Lawson et al. (2017) successfully explained their ASD group’s reduced reliance on prior information by a hierarchical Bayesian-learning model^1^; critically, this computational model assumed that ASD individuals “overestimate the volatility of the sensory environment”, as a result of abnormally high weighting of prediction errors regarding environmental volatility, and consequently fail to build stable expectations (but the generality of this account has been challenged, see Manning et al. 2017).

### Atypical Predictive Coding in Pre-attentive, as well as Attentional, Vision?

In all of the studies mentioned above that used visual classification tasks (Lawson et al. 2017; Skewes et al. 2014), there was only one stimulus in the display, and this was task-relevant and, thus, focally attended. However, this scenario captures only part of how we deal with complex visual scenes, where a pre-attentive system of ‘priority’ computations determines where to allocate attention and post-selective perceptual processes then decide what the object is that has been brought into the focus of attention (e.g., Koch and Ullman 1985; Wolfe 2007). In other words, decisions about where to attend precede perceptual decisions, and the former can also be influenced by prior knowledge—as evidenced by the emergent literature on the guidance of visual search by statistically learnt ‘context’ cues (e.g., Chun and Jiang 1998) or acquired knowledge about where in a scene potentially distracting stimuli are likely to occur (Sauter et al. 2018). Given this, the present study was designed to establish whether the tendency of ASD individuals to rely less on prior knowledge, as compared to TD individuals, would also apply to decisions about where to attend at the pre-selective stage, as well as to perceptual decisions about objects in the focus of attention at the post-selective stage.

### Rationale of the Present Study

To this end, we used a variant of the ‘additional-singleton’ visual-search paradigm (Theeuwes 1992), in which participants search for and respond to a target singleton—typically an item of a unique shape—, while ignoring a more salient but task-irrelevant distractor singleton—typically an item of a unique color. In our variant of this paradigm, the to-be-ignored distractor was more likely to appear in one specific display region (e.g., the upper region), while the target appeared with equal likelihood at all possible locations—that is, the likely distractor region was probabilistically ‘cued’. Prior work has shown that TD participants can acquire this cue by statistical learning and use it to ‘proactively’ (e.g., Geng 2014) and tonically suppress salient distractors occurring at the likely, or ‘frequent’, positions, as evidenced by such distractors generating less reaction-time (RT) interference—indicative of less ‘involuntary attentional capture’—compared to distractors occurring at unlikely, or ‘rare’, locations (Allenmark et al. 2019; Sauter et al. 2018; Wang and Theeuwes 2018a; Zhang et al. 2019).

If individuals with ASD rely less on prior knowledge when deciding which item in the search display to attend to, one would expect this to be reflected in a diminished difference in interference between distractors appearing in the frequent vs. the rare distractor region. However, instead of being due to the prevention of attentional capture at the pre-selective stage, reduced RT interference from distractors at the frequent (vs. rare) locations that is found with TD individuals could also arise because, following mis-allocation of attention, and the eye gaze, to this location, they may have learned to more efficiently decide, at the post-selective stage, that the stimulus at this location is a task-irrelevant distractor rather than the response-relevant target. This would permit them to disengage attention, and the eye, faster from this location and reallocate it to the target location, and so expedite the final RTs. Thus, if individuals with ASD were found to make less use of statistical distractor-location cues, this could also be attributable to them relying less on prior knowledge when making a perceptual classification about whether an attended (fixated) item is a distractor rather than a target, during the post-selective decision stage. To disentangle these possibilities, we collected eye-tracking data in addition to manual RT data in our variant of the additional-singleton paradigm. An influence of prior knowledge during the pre-selective stage can be assessed based on the eye-movement data, in terms of the proportions of mis-guided first saccades to/fixations on the distractor—which would be expected to be lower for distractors at the frequent versus the distractor locations; and influences during the post-selective stage can be assessed from the durations of fixations on the distractor—(which would be expected to be shorter on distractors at the frequent vs. the rare locations; see, e.g., Wang et al. 2019).

Note that in the additional-singleton paradigm, the target and distractor locations, by design, never coincide on a given trial *n*, though the target can appear at the position of a distractor on the previous trial *n* – 1. If a distractor captures attention, its location needs to be suppressed ‘reactively’ (e.g., Geng 2014) in order for attention to be disengaged and reallocated to the target location, and this inhibition carries over to the next trial—as evidenced by slowed RTs to a target on a given trial *n* falling at the location occupied by a distractor on the preceding trial *n* – 1 (e.g., Geyer et al. 2006; Kumada and Humphreys 2002; Maljkovic and Nakayama 1996; Sauter et al. 2018). Henceforth, such trials will be referred to as ‘coincidence trials’. Applied to the paradigm variant with distractor-location probability cueing, a distractor occurring at an unlikely location—which is not subject to learnt, proactive suppression—would be more likely to capture attention compared to a distractor at a likely location. Such a rare-location distractor would be less expected and so have greater surprise value when it captures attention—setting in motion reactive suppression and short-term prior updating processes, affecting performance on subsequent trials.

If individuals with ASD have difficulty with down-weighting irrelevant prediction errors, as assumed by the precision-regulation account, they may sometimes overreact to unexpected events. Applied to the present paradigm, they may be expected to respond to rare-location distractors with more reactive suppression and/or other short-term prior updating processes. This would affect performance on the subsequent trials. Specifically, when the target occurs at the re-actively suppressed location, individuals with ASD may exhibit a larger cost on rare-location coincidence trials, as compared to TD individuals. By contrast, the costs may not differ, or differ by less, for frequent-location coincidence trials, that is, when the target occurs at a proactively suppressed location. Thus, by examining the general distractor-location probability-cueing effect and the coincidence costs, we aimed to gain insight both into how ASD individuals use priors during the pre-attentive stage of visual search, and how they update priors in response to unexpected events.

## Methods

### Participants

Twenty-two adults on the autism spectrum [nine female; age range 18–67 (mean = 30.4, SD = 13.5) years] and 22 TD adults [nine female; age range 18–70 years (mean = 29.7, SD = 12.9)] were recruited from the database of the Outpatient Clinic for Autism Spectrum Disorders at the Department of Psychiatry, LMU Munich, as well as through local autism network contacts. TD participants were recruited from the database of the LMU Faculty of Psychology and Pedagogics, which contains a large number of individuals available to take part in psychological studies (either psychology students, people generally interested in psychological studies, or people just wishing to earn some extra money by participating in psychological studies) or among local medical students. The TD participants were selected to, as closely as possible, match the distribution of gender and age of the group of ASD participants An additional selection criterion was that participants had to be fluent in German (since the questionnaires were in German). IQ was not used as a selection criterion, but the mean IQs still ended up closely matched between the two groups.

All ASD participants had been diagnosed by a team of certified psychologists and psychiatrists according to the (American Psychiatric Association 2013) ICD-10 (WHO 1992). None of the TD participants reported any history of mental illnesses or neurological deficits.

The sample size needed for 90% power was calculated based on the effect sizes of the distractor-location effects in two previous probability-cueing studies using a similar paradigm: the study of (Wang and Theeuwes 2018a) and our replication of this study (Zhang et al. 2019). Since our previous study had two sessions, we calculated the effect size based on the first session only: our effect size was comparable to that reported by Wang and Theeuwes but slightly smaller (d_z_ = 1.8 compared to d_z_ = 2.0), and so we conservatively based our power calculation on that, smaller effect size. We then calculated the sample size required for detecting a between-group difference with 90% power, with one-tailed testing, assuming the effect size from our previous study in the TD group and half as large an effect in the ASD group (this is roughly the group difference found by Lawson et al. 2014) as 22 participants.

Participants with and without ASD were pairwise matched on the ‘Wortschatztest’ (WST, Schmidt and Metzler 1992) IQ-Scale (ASD group mean = 105.86; TD group mean = 105.91), gender, and age. Both groups completed the Autism-Spectrum Quotient (AQ, Baron-Cohen et al. 2001), Empathy Quotient (EQ, Baron-Cohen and Wheelwright 2004), Systemizing Quotient (Baron-Cohen et al. 2003), Beck’s Depression Inventory (BDI, Beck et al. 2011). The ASD and TD groups did not differ significantly in terms of age or IQ. But there were significant differences between the two groups in the AQ, EQ, and SQ measures (see Table 1).

**Table 1:** Descriptive characteristics of the ASD and TD groups

Measures	ASD group (*n* = 22)	TD group (*n* = 22)	Group comparison	Significance
AGE	30.4 (13.5)	29.7 (12.9)	*t*(42) = 0.17, *p* = 0.86, *BF*_10_ = 0.3	
IQ	105.9 (10.8)	105.9 (11.7)	*t*(42) = − 0.01, *p* = 0.99, *BF*_10_ = 0.3	
Autism-spectrum quotient score	36.5 (7.2)	15.5 (6.5)	*t*(42) = 10.2, *p* < 0.001, *BF*_10_ > 1000	***
Empathy quotient score	28.6 (13.1)	52.8 (15.2)	*t*(42) = − 5.6, *p* < 0.001, *BF*_10_ > 1000	***
Systemizing quotient score	35.5 (16.6)	25.5 (8.4)	*t*(42) = 2.5, *p* < 0.05, *BF*_10_ = 3.5	*
Beck’s depression inventory score	8.9 (6.23)	5.0 (6.39)	*t*(42) = 2.1, *p* < 0.05, *BF*_10_ = 1.6	*

Numbers in brackets are standard deviations

*Denotes *p* < 0.05

***Denotes *p* < 0.001

The study was approved by the Ethics Board of the LMU Department of Psychology. All participants gave written informed consent prior to commencing the experiment and received 10 Euros per hour for their service.

### Apparatus

The experiment was carried out in a sound-reduced and moderately lit experimental cabin. The stimuli were presented on a 21-inch LACIE CRT monitor with a screen resolution of 1024 × 768 pixels and a refresh rate of 85 Hz. Movements of participants’ dominant eye were monitored using an Eyelink 1000 desktop-mounted system (SR Research, Canada), set at a sampling rate of 1 kHz. Stimulus presentation, response recording, and eye-movement sampling were controlled via a customized Matlab program using the Psychtoolbox and the Eyelink Toolbox (Brainard 1997).

### Stimuli

**Fig. 1:**
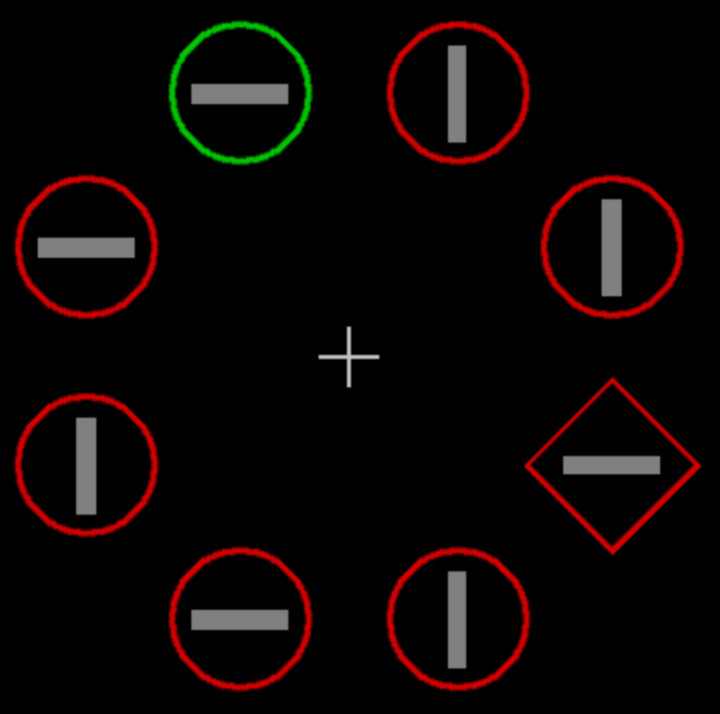
Example search display. The search target is the singleton shape (here the *diamond* shape), and the distractor is a color singleton (here the *green* item). Participants responded to the orientation of the bar inside the target shape (here *horizontal*)

The search displays (see Fig. 1) were composed of eight colored outline shapes (circles or diamonds), arranged equidistantly around a virtual circle (radius of 4.4° of visual angle). The display items consisted of either one circle (target) and seven diamonds (non-targets), or, alternatively, one diamond (target) and seven circles (non-targets). In a certain percentage of trials, one of the non-target shapes (the distractor) differed in color from all the other shapes, being either green amongst homogeneous red shapes, or red amongst green shapes. All search displays were presented on black screen background, with a white fixation cross in the center. Each outline shape contained a vertical or horizontal gray line inside (0.3° × 1.5°). On each trial, half of the internal lines, randomly chosen, were vertical and half horizontal.

### Design

On each trial, one of the eight shapes in the search display was a singleton shape, either a circle among diamonds or a diamond among circles, randomly assigned on each trial. Participants had to find this—target—shape and respond to the orientation of the line inside it. In 66% of trials, there was also a uniquely colored singleton distractor, a single red amongst green shapes or green amongst red shapes, randomly assigned on each trial. The target appeared at each location with equal probability, but never at the same location as the distractor on a given distractor-present trial. The distractor, on the other hand, appeared with 90% probability in one—the top or bottom—half of the search display, the frequent region, and with 10% probability in the other half, the rare region (i.e., we adopted a variant of the region cueing paradigm pioneered by Goschy et al. 2014). Which region was frequent and which rare was counterbalanced across participants within each group. Within each region, the distractor appeared at each of the four locations with equal probability. Participants performed 1020 trials overall, divided into 17 blocks of 60 trials each.

### Procedure

Each trial started with the presentation of a fixation cross for a random duration between 1.2 and 1.45 s. This was followed by the search display which was shown until the participant responded. Participants were instructed to search for the uniquely shaped ‘target’ object and identify the orientation of the line contained inside it, vertical or horizontal, as quickly and accurately as possible. They were instructed to press the ‘up’ arrow on the keyboard if the line was vertical, and the ‘left’ arrow if it was horizontal. If a participant gave an incorrect response, the feedback message “incorrect response” was displayed for 500 ms, followed by 300 ms with a blank screen before the start of the next trial.

### Bayes-Factor Analysis

Bayesian analyses of variance (ANOVAs) were performed using JASP (http://www.jasp-stats.org) with default settings (i.e., r-scale fixed effects = 0.5, r-scale random effects = 1, r-scale covariates = 0.354). All Bayes factors reported for ANOVA main effects and interactions are “inclusion” Bayes factors calculated across matched models. Inclusion Bayes factors compare models with a particular predictor to models that exclude that predictor, providing a measure of the extent to which the data support inclusion of a factor in the model. Bayesian *t* tests were performed using the ttestBF function of the R package “BayesFactor” with the default setting (i.e., rscale = “medium”).

## Results

The Results section is organized into two parts: The first reports the full manual-RT analysis, which reveals a certain, strikingly altered characteristic of ASD individuals’ response behavior, but offers no explanation of how this alteration is brought about. The second part presents an analysis of the eye-movement data, with particular focus on the oculomotor dynamics in the critical, altered condition, effectively testing, and deciding between, alternative accounts of the underlying cause of altered perceptual decision-making in individuals with ASD.

### RT Results

All RT analyses were performed on individuals’ median RTs after excluding trials on which a participant made an incorrect response (approximately 3% of trials on average).

**Fig. 2:**
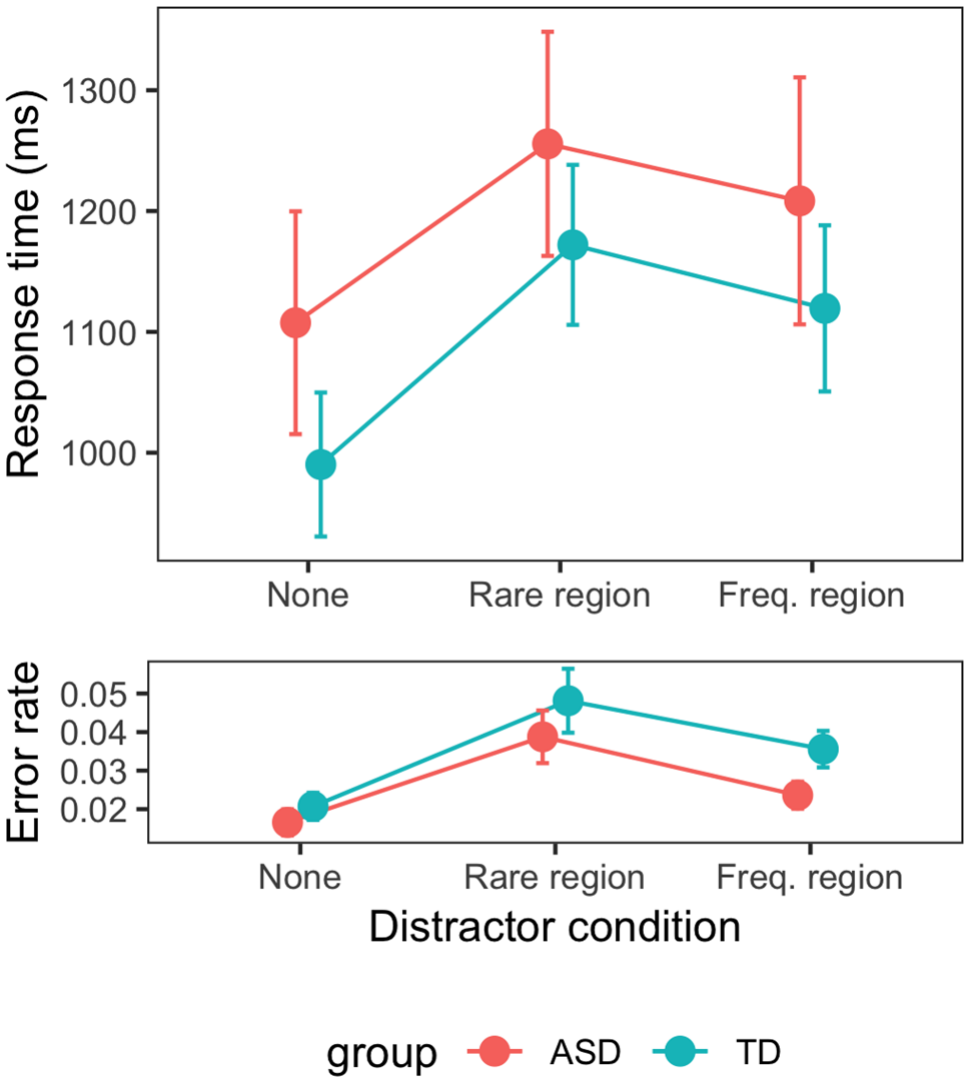
RTs and error rates as a function of distractor condition (none: distractor absent, rare region: distractor in rare region, freq. region: distractor in frequent region), separately for the ASD and TD groups. *Error bars* denote one standard error

Figure 2 depicts the RTs, and error rates, for the different distractor conditions (distractor absent, distractor in rare region, distractor in frequent region), separately for the ASD and the TD group. Numerically, RTs were slower in the ASD than in the TD group (distractor-present trials: 1173 vs. 1076 ms; distractor-absent trials: 1108 vs. 990 ms). However, a mixed-design ANOVA with the factors distractor condition and group failed to reveal this difference to be significant [main effect of group: *F*(1, 42) = 0.74, *p* = 0.39, η_p_^2^ = 0.017, *BF*_incl_ = 0.67]. Importantly, the main effect of distractor condition was significant, *F*(2, 42) = 101.53, *p* < 0.001, η_p_^2^ = 0.71, *BF*_incl_ > 1000, but not the interaction with group, *F*(1,42) = 1.17, *p* = 0.32, η_p_^2^ = 0.03, *BF*_incl_ = 0.37.

To break down the distractor-condition effect, we analyzed (1) the overall amount of distractor interference, defined as the RT difference between distractor-present trials (averaged with equal weight for trials with a distractor in the rare and, respectively, the frequent region) and distractor-absent trials, and (2) the distractor-location (*probability-cueing)* effect, defined as the RT difference between trials with a distractor in the rare and, respectively, the frequent region (Sauter et al. 2018; Zhang et al. 2019). Distractor interference was somewhat lower in the ASD group (124 vs. 156 ms in the TD group), but the difference was not significant [Welch two-sample *t* test: *t*(38.01) = − 1.49, *p* = 0.14, *BF*_10_ = 0.72]. Importantly, there was a significant probability-cueing effect [50 ms, *t*(43) = 4.31, *p* < 0.001, *BF*_10_ = 253], which did, however, not differ significantly between the ASD and TD groups [47 vs. 53 ms; Welch two-sample *t* test: *t*(40.77) = − 0.23, *p* = 0.83, *BF*_10_ = 0.30]; in fact, the Bayesian analysis provides substantial evidence in favor of no difference. Thus, the individuals with ASD learnt to proactively suppress distractors in the frequent distractor region as effectively as TD individuals—at variance with the former being compromised in their ability to acquire the distractor-distribution ‘prior’ (i.e., with the hypo-prior account).

#### Target-Position Effect

**Fig. 3:**
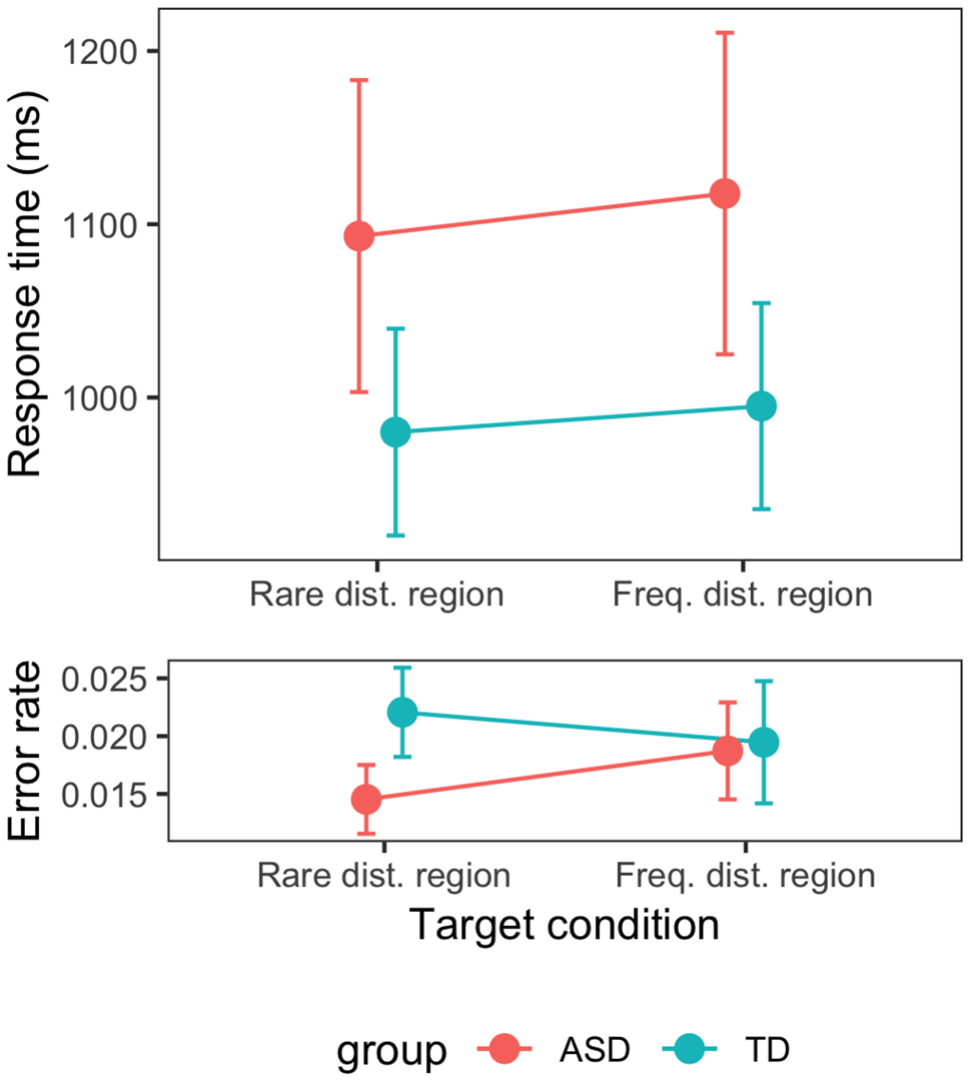
RTs and error rates as a function of the target condition (target in rare vs. frequent distractor region), separately for the ASD and TD groups. *Error bars* denote one standard error

**Fig. 4:**
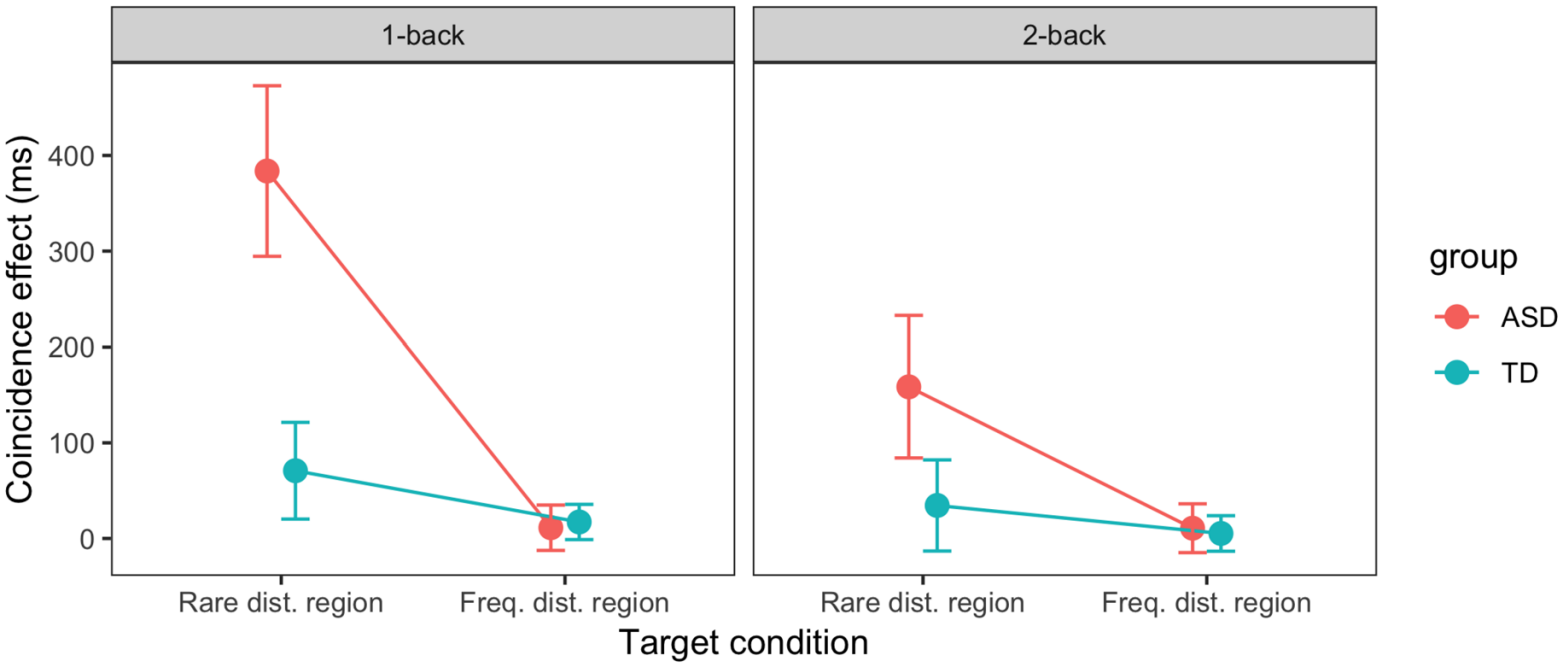
Effect of spatial coincidence of the distractor position on trial *n* − 1 (*left*) or *n* − 2 (*right*) and the target position on trial *n* (i.e., the difference in RTs between distractor-absent trials *n* on which the current target did vs. did not appear at the location of the distractor on the preceding trial *n* − 1 or *n* − 2) as a function of the target location on trial *n* (in frequent vs. rare distractor region), separately for the ASD and TD groups. *Error bars* denote one standard error

Figure 3 depicts the RTs, and average error rates, on distractor-absent trials, depending on whether the target appeared in the rare or the frequent distractor region, for the two participant groups (ASD and TD). For this analysis, trials on which the target appeared at the same location as a distractor on the previous trial were removed, to rule out confounding of the target position effect by carry-over of inhibition of the distractor location on the preceding trial (see coincidence effects depicted in Fig. 4). A mixed-design ANOVA with the factors target condition and group revealed RTs to be significantly slower for targets appearing in the frequent versus the rare distractor region [1055 vs. 1035 ms; *F*(1, 42) = 4.67, *p* = 0.036, η_p_^2^ = 0.10, *BF*_incl_ = 1.67]—replicating previous studies using this paradigm (Wang and Theeuwes 2018a, b; Zhang et al. 2019). However, this target-position effect did not differ significantly between the ASD and TD groups [25 vs. 15 ms; *F*(1, 42) = 0.28, *p* = 0.60, *BF*_incl_ = 0.34]—indicating that both groups acquired the same strategy of generally suppressing any singleton targets and distractors in the frequent distractor region, in line with suppression operating at the level of the search-guiding priority map (see Liesefeld and Müller 2019, 2020; Sauter et al. 2018, 2019; 2020).

#### Carry-Over of Reactive Distractor Inhibition Across Trials

Given this, we went on to test the alternative hypothesis that individuals with ASD react more strongly to rare, as compared to frequent, distractor events—by examining carry-over of reactive inhibition placed on the distractor location on a given trial onto targets appearing at the same location on the subsequent trial. That is, we analyzed the distractor_*n*–1_–target_*n*_ position coincidence trials, focusing exclusively on distractor-absent trials *n* (to assess pure carry-over of inhibition uninfluenced by any dynamics set in motion by the presence of a distractor on trial *n*; see Zhang et al. 2019). Figure 4 presents the distractor–target coincidence effect—the difference in RTs between distractor-absent trials *n* on which the current target did versus did not appear at the location of the distractor on the preceding trial *n* – 1—separately for the two groups.

A mixed-design ANOVA with target condition (target *n* in frequent vs. rare distractor region) and group (ASD vs. TD) as factors revealed both main effects to be significant [target condition: *F*(1, 42) = 17.02, *p* < 0.001, η_p_^2^ = 0.29, *BF*_inc_ > 1000; group: *F*(1, 42) = 7.75, *p* = 0.008, η_p_^2^ = 0.16, *BF*_inc_ = 53]; importantly, the interaction also turned out significant, *F*(1, 42) = 9.53, *p* = 0.004, η_p_^2^ = 0.19, *BF*_inc_ = 76. The distractor–target coincidence effect (i.e., the carried-over inhibition) was substantial and significant only for targets in the rare region [228-ms effect, *t*(43) = 4.1, *p* < 0.001, *BF*_10_ = 124; frequent region: 14-ms effect, *t*(43) = 0.96, *p* = 0.34, *BF*_10_ = 0.25]. Further, it was equally small (and non-significant) for both groups if the current target coincided with a preceding distractor in the frequent region (ASD: 11-ms effect; TD: 17-ms effect); by contrast, for targets following a distractor in the rare region, the effect was more than five times larger for ASD than for TD individuals (384 vs. 71 ms, Welch two sample *t* test: *t*(33.2) = 3.05, *p* = 0.004, *BF*_10_ = 10). The negligible coincidence effect for targets in the frequent region is as expected: this region is proactively (tonically) suppressed, curtailing any additional re-active (phasic) inhibition in response to an actual distractor occurring there (Zhang et al. 2019). Similarly, as the rare distractor region is not tonically suppressed, the increased coincidence effect for this region is also as expected. However, the size of the group difference is striking: individuals with ASD display what looks like a ‘qualitative’ increase in reactive inhibition to unexpected distractors. This effect is not a chance finding: we still find a trace of it on the next trial,^2^ when the spatially coincident distractor (with the current target) occurred two trials back (distractor_*n–2*_–target_*n*_): here, a significant 159-ms effect for individuals with ASD [*t*(19) = 2.1, *p* = 0.046, *BF*_10_ = 1.5] compares with a 35-ms effect for TD individuals.

#### Interim Discussion

Thus, the only, and striking, difference between the ASD and TD groups is that, in ASD, the coincidence effect was considerably larger, by a factor of at least five, when the target appeared at a previous distractor location, but only if the target and the previous distractor appeared in the rare distractor region. There are two possible explanations for this pattern: (1) Stronger reactive inhibition placed on—or ‘inhibition of return’ (IOR, Klein 2000; Posner and Cohen 1984) to—a previous distractor location in the rare region, which would be consistent with reports of stronger IOR in individuals with ASD in a Posner-type cueing paradigm (Rinehart et al. 2008). Or (2), a reactively strengthened belief that any salience signal (indicative of the presence of a singleton item) arising at the same location as the distractor in the rare region on the previous trial must actually be caused by a distractor, rather than a target. From the perspective of a drift–diffusion model (e.g., Ratcliff et al. 2016), there may be a reactively strengthened decision bias towards identifying the item at such a location as a ‘distractor’, and against identifying it as a response-relevant ‘target’—involving, say, a shift of the starting point of the evidence accumulation process closer to a ‘distractor’ decision and away from a ‘target’ decision. Although appearing similar to account (1), account (2) is subtly different: it is not the allocation of attention to the previous distractor location in the rare region as such that is altered (i.e., more strongly inhibited), but the amount of evidence that needs to be accumulated by focal-attentional processing of the item at this location to arrive at a target decision.

Deciding between these alternatives is not possible based on the RT results alone, as RTs represent only the end of a whole chain of processes leading up to the final response. However, we can gain insight into this chain by analyzing the patterns of eye movements on a given (type of) trial. In what follows, we summarize the main findings of the eye-movement analysis, following prior eye-movement studies of attentional capture (Di Caro et al. 2019; Geyer et al. 2008; Wang et al. 2019), though with a focus on the critical (ASD vs. TD differential) RT effects in the present study. Details of the eye-movement analyses and findings are presented in Supplementary.

### Eye-Movement Results

Previous ‘attentional-capture’ studies of distractor-location probability-cueing effects using similar stimuli to the present study have found a reduced likelihood of oculomotor capture by distractors at frequent versus rare distractor locations, and potentially expedited disengagement of the eye from a distractor that had summoned a saccade at frequent versus rare locations. Also, on distractor-absent trials, saccade latencies to targets appearing at the frequent distractor location were delayed (Wang et al. 2019). Overall, this is consistent with the view that distractor-location probability learning involves proactive inhibition of the likely distractor locations, and, possibly, faster disengagement of attention, and the eye, from distractors at these locations. Accordingly, we first examined whether the ASD and TD groups would show similar effect patterns on distractor-present and -absent trials.

#### Distractor-Location Effects on Distractor-Present Trials

Both participant groups required overall fewer eye movements to acquire the response-critical target information with a distractor present in the frequent versus the rare distractor region [3.76 vs. 3.91 fixations, *F*(1, 37) = 10.11, *p* < 0.01; this effect did not significantly interact with group]. The main reason for this was that oculo-motor capture of the first saccade by the distractor was reduced [25 vs. 35%, *F*(1, 37) = 9.90, *p* < 0.01] and, in a trade-off, first eye movements going straight to the target were increased with a distractor in the frequent, versus one in the rare, region [28 vs. 21%; *F*(1, 37) = 38.01, *p* < 0.001],^3^ for both groups. This pattern is consistent with learnt proactive inhibition of the frequent distractor region. Further, the first distractor fixations were marginally shorter on distractors that appeared in the frequent region, as compared to distractors in the rare region [203 vs. 213 ms: *F*(1, 36) = 3.82, *p* = 0.06]. This indicates that disengagement of attention from a distractor in the frequent region was expedited. Thus, replicating Wang et al.’s (2019) prior study with unimpaired young participants, both proactive inhibition of locations in the frequent distractor region, preventing oculo-motor capture by a distractor in the first instance, and, if capture prevention failed, expedited disengagement of the eye from a distractor in the frequent region contribute to the acquired (overall) RT probability-cueing effect. This is the case equally for the ASD and the TD group.

#### Distractor_n−1_–Target_n_ Spatial-Coincidence Effect on Distractor-Absent Trials

On coincidence trials, the percentage of first saccades directed straight to the target tended to be increased overall for targets following a distractor in the rare distractor region [an additional 9% as compared to non-coincident trials, vs. an additional 1% in the frequent region, *F*(1, 35) = 2.71, *p* = 0.11]. If anything, this effect was stronger in individuals with ASD (an additional 13% in the rare region vs. 1% in the frequent region); that is, compared to TD individuals (an additional 5% in the rare vs. 2% in the frequent region), a greater percentage of their first saccades went directly to a target located in the rare region [rare region, coincident vs. non-coincident trials: *t*(19) = 2.07, *p* = 0.052].^4^ The increased proportion of first saccades directed to the target effectively rules out increased cross-trial IOR of the preceding distractor location as an explanation for why ASD individuals took so long to respond to a target that followed a distractor at the exact-same (‘coincident’) location in the rare region. Increased IOR would have greatly reduced the ‘priority’ of the rare distractor location, decreasing the likelihood of attention, and the eye, returning there on trial on trial *n*. However, if anything, a target at this location was more, rather than less, likely to attract the first saccade, which is inconsistent with a—due to cross-trial IOR—lowered attention-attracting power of this item in ASD versus TD individuals.

However, while individuals with ASD were somewhat more likely to direct their first saccade to the target in the rare distractor region, the average number of fixations they took to make a response decision on coincidence, relative to non-coincidence, trials tended to be increased when the target appeared in the rare region [3.93 vs. 3.29 fixations, *t*(19) = 1.89, *p* = 0.07], but not for targets in the frequent region [3.42 vs 3.35 fixations, *t*(19) = 0.70, *p* = 0.49]; for TD individuals, by contrast, the number of fixations (on coincidence vs. non-coincidence trials) was comparable with targets in the rare region [3.31 vs. 3.33 fixations, *t*(16) = − 0.08, *p* = 0.93], as well as targets in the frequent region [3.41 vs. 3.44 fixations, *t*(16) = − 0.36, *p* = 0.72]. Thus, individuals with ASD required more eye movements to arrive at a response decision when the target followed a distractor at the ‘coincident’ location in the rare region, accounting for their slowed RTs on such coincidence trials. This is the case even though their first saccade was, if anything, more likely to be directed straight to the target.

Given that the first eye movement cannot account for the increased ‘coincidence’ effects with spatially coincident distractors and targets in the rare region, we conducted a more detailed analysis of the extended oculomotor scanning patterns on such trials to uncover why individuals with ASD require more eye movements. In particular, we partitioned participants’ scanning behavior on all distractor-absent trials into three categories based on the ‘target fixation pattern’ (treating multiple fixations in a row on the target as a single target fixation, in each category). Category 1 consisted of trials on which the target was fixated a single time before response, henceforth referred to as ‘*single final target fixation*’ trials; category 2: trials on which the target was fixated a single time, followed by multiple fixations on other items before response, ‘*single non-final target fixation*’ trials; and category 3: trials on which the target was fixated more than once, with at least one fixation of a non-target item in between, ‘*target re-fixation*’ trials.

Collapsed across coincident and non-coincident trials, the total dwell time spent fixating the target was longest in the ‘single final’ condition for both groups, and similarly short in the ‘single non-final target fixation’ and ‘target re-fixation’ conditions, *F*(2, 62) = 23.0, *p* < 0.001. At the same time, the behavioral RT was shortest in the ‘single final’ condition, and longest in the ‘target re-fixation’ condition, with the ‘single non-final’ condition in between [*F*(2, 62) = 123.9, *p* < 0.001]. The slow RTs in the ‘target re-fixation’ condition and the relatively slow RTs in the ‘single non-final target fixation’ condition likely reflect the fact that, on the first visit to the target, the target was mis-identified as a non-target/distractor, as a result of which oculomotor inspection moved elsewhere before either returning to the target or its vicinity to extract the response-relevant information (here, the orientation of the bar inside the target shape). Zhaoping and Guyader (2007) described a similar oculomotor pattern in a study of saliency-driven attention allocation in standard visual search in the absence of salient distractors: even though the target, as the most salient item, attracted the first eye movement, oculomotor scanning went on to other, non-target items before eventually returning to the target and responding.

**Fig. 5:**
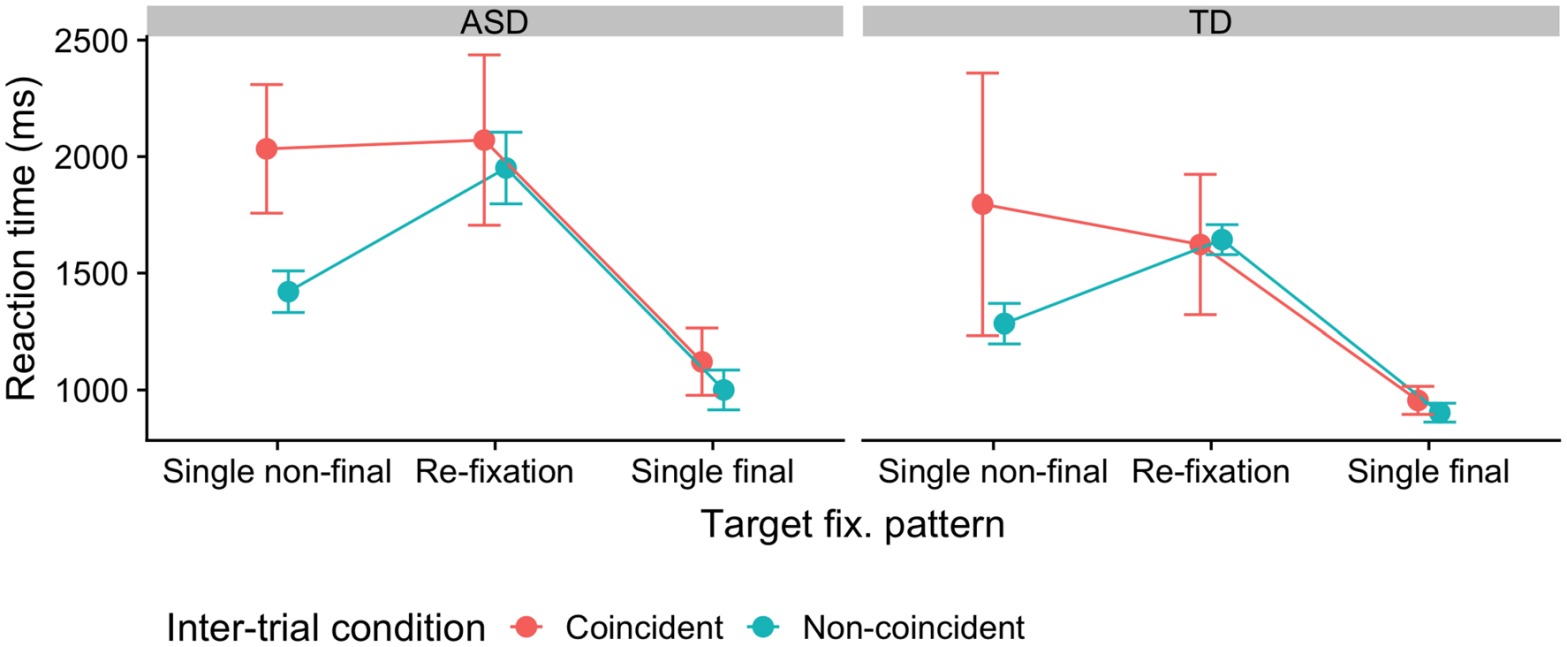
RTs on distractor-absent trials on which the target appeared in the rare distractor region, for coincident (target appearing at previous distractor location) and non-coincident trials (target appearing at non-distractor location), separately for the different target fixation patterns

Looking at the RTs as a function of the fixation pattern with a target in the rare distractor region, and comparing the critical coincident versus non-coincident trials (Fig. 5), RTs do not differ much between coincident and non-coincident trials in either the ‘single final’ (1038 vs 952 ms) or the ‘target re-fixation’ condition (1847 vs 1797 ms); they only differ in the ‘single non-final target fixation’ condition, where there is a substantial cost in responding to coincident, relative to non-coincident, targets (1915 vs 1352 ms). The reason for this may be that after sampling the target and mis-identifying it as a distractor or non-target, it took longer to accumulate information about the response-critical target feature without returning to the target (i.e., from eccentric vision). Since both groups show essentially the same pattern, this cannot explain why, in the ASD group, responses were particularly slow to coincident targets in the rare distractor region.

**Fig. 6:**
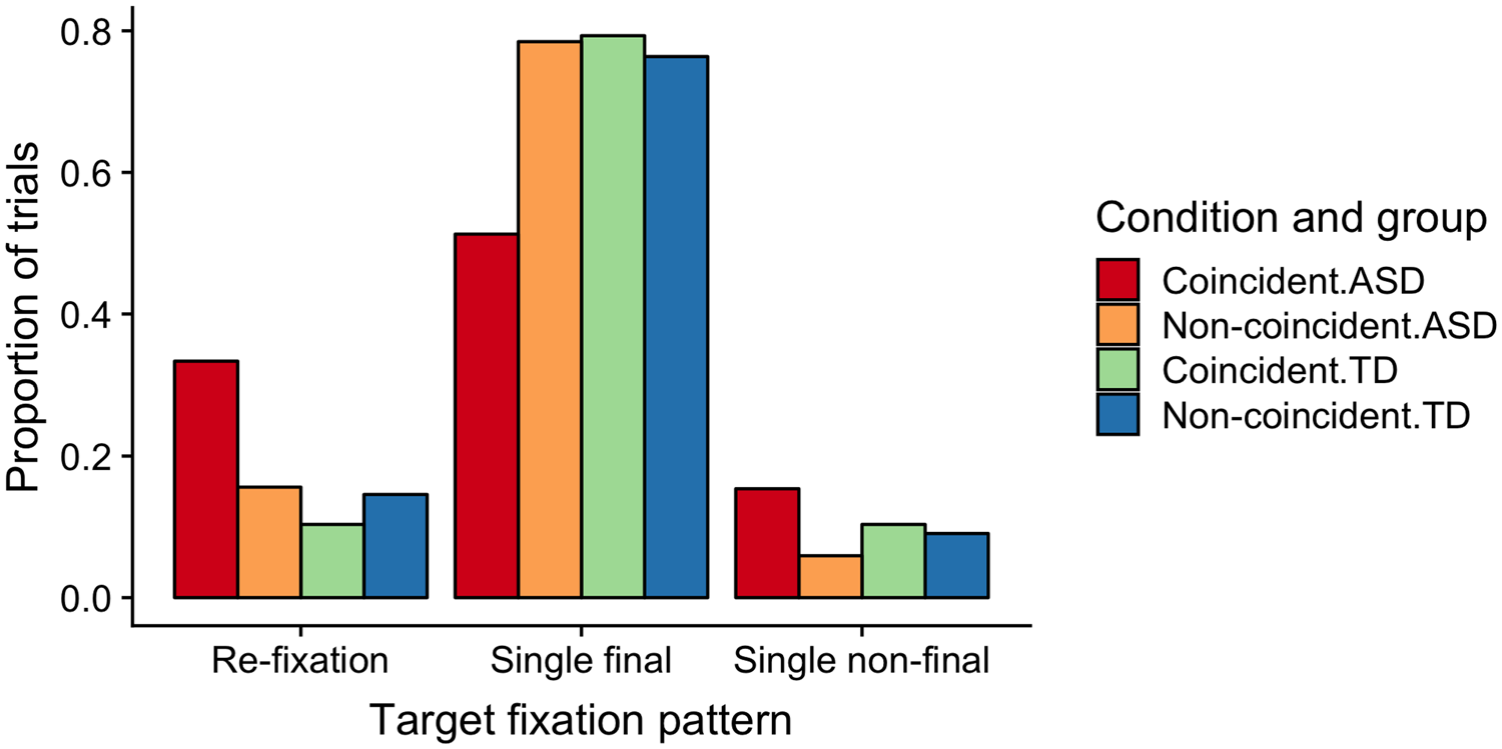
Proportions of trials with the different target-fixation patterns. Proportions depicted for (distractor-absent) trials on which the target appeared in the rare distractor region, separately for coincident (target at previous distractor position) and non-coincident target locations (target at previous non-distractor location), separately for the ASD and TD groups

Accordingly, since the reaction- and dwell-time measures did not differ between the two participant groups, the explanation for this differential pattern must lie in the frequencies with which ASD and, respectively, TD individuals produced a particular eye-movement pattern. Indeed, examining the proportions of distractor-absent trials on which the target appeared in the rare distractor region, with the different target-fixation patterns (see Fig. 6) revealed the frequencies of these patterns to differ between coincident and non-coincident trials for the two groups: Both groups showed near-equivalent frequencies of the three target-fixation patterns on non-coincident trials (‘single-final target fixations’ being most frequent, at 77%, and ‘single non-final’ and ‘target re-fixation’ patterns being relatively infrequent, at 8 and 15%, respectively). However, in the ASD group, the percentage of trials with the ‘single final’ pattern was reduced on coincident, as compared to non-coincident, trials and those with the ‘single non-final’ and, most markedly, the ‘target re-fixation’ patterns were increased (51% ‘single final’, 15% ‘single non-final’, and 33% ‘target re-fixation’). No such change in the frequencies of target-fixation patterns (for coincident vs. non-coincident trials) was evident in the TD group (79% ‘single final’, 10% ‘single non-final’, and 10% ‘target re-fixation’). Chi-square tests revealed the distributions of target-fixation patterns to differ significantly between coincident and non-coincident trials in the ASD group [Χ^2^(2, *N* = 1525) = 16.6, *p* < 0.001], but not the TD group [Χ^2^(2, *N* = 1607) = 0.44, *p* = 0.80].

#### Interim Discussion

This effect pattern suggests that the main reason for why individuals with ASD took so long to make a response to a target (on trial *n*) appearing at the previous (trial *n* − 1) distractor location in the rare region is that, although their eyes were initially attracted to the singleton target as efficiently as in TD individuals, they then moved away from the target before eventually returning there (‘target re-fixation’ trials) or its vicinity (‘single non-final target fixation’ trials) and making the response decision. This is inconsistent with IOR-based accounts, whether they assume cross-trial IOR to be increased for the distractor location on the preceding trial *n* − 1 or within-trial IOR for the location first inspected on the current trial *n*. At variance with increased cross-trial IOR for the preceding distractor location, the (trial *n*) target at this location did attract the eye as rapidly in ASD as in TD individuals; and at odds with increased within-trial IOR, ASD individuals returned nearly as readily as TD individuals to the ‘coincident’ target location after having inspected it first (i.e., on “target re-fixation” trials).

Thus, instead, this pattern is likely to reflect a carried-over (from the preceding trial) bias against making a ‘target’ decision to the item occupying the location of the preceding distractor in the rare region. That is, for individuals with ASD, having detected a distractor at this location on trial *n* − 1 disproportionately strengthens their prior belief that this location contains a distractor on the subsequent trial *n*. As a result, when first attending to/fixating the ‘coincident’ target location on trial *n*, they tend to mis-identify it as a distractor. Consequently, their search is redirected elsewhere and returns only later, after not having found the target at other display locations, to the ‘coincident’ target position.

## General Discussion

The present study was designed (1) to examine at which processing stage(s) predictive coding is altered in ASD: at a pre-selective stage that decides which objects to bring into the focus of attention, and/or a post-selective stage that decides what the attended stimulus is; and (2) to determine exactly in which way perceptual decision processes are altered by abnormal predictive coding in ASD. To this end, we combined an additional-singleton visual-search task with a manipulation of the probability with which a salient but task-irrelevant distractor occurred in a particular display half. This paradigm had previously been used to demonstrate that participants can learn from experience to more efficiently prevent attentional capture by a salient singleton distractor when this occurs in a frequent, as compared to a rare, distractor region (Sauter et al. 2018; Wang and Theeuwes 2018a, b; Zhang et al. 2019). Consistent with these studies, we found that search performance, measured by the manual RTs to the target, was less affected by the presence of an irrelevant distractor when it appeared in the frequent region, indicative of proactive suppression of this region. This probability-cueing effect was not significantly reduced in the ASD, compared to the TD, group; rather, both groups learnt and made use of the bias in the spatial distractor distribution (i.e., the ‘prior’) equally efficiently. Accordingly, at least with respect to distractor-location probability learning, it is not the acquisition of prior beliefs that is compromised in individuals with ASD, at variance with ‘hypo-prior’ accounts (Pellicano and Burr 2012) of the reduced reliance on prior information in ASD. An alternative possibility is that the autistic cognitive profile is characterized by altered reactions to unlikely events (signaling changes) in the environment, in line with precision-regulation accounts (Lawson et al. 2017). As we will argue below, altered precision weighting can explain the main finding of the present study, namely, that, compared to TD individuals, ASD individuals required substantially more time to respond to the target when it appeared at a previous distractor location, but only if the target fell on the same location as the preceding distractor in the rare distractor region.

One possible interpretation of this positional ‘distractor–target coincidence’ effect is that it reflects reactive, IOR-type inhibition placed on the distractor location, after the distractor had captured attention, in order to disengage attention from the distractor and reorient it to the target, and to generally prevent attention from returning there; this inhibitory effect could be carried over across trials (see also Klein 2000; Rinehart et al. 2008). However, this interpretation was effectively ruled out by our eye-tracking results, which showed that ASD individuals, if anything, were *more* likely to look at the target before any other item in the search display when it appeared at a previous distractor location in the rare region. Instead, the large coincidence effect in the ASD group originated from an increased proportion of trials on which a participant, after the first visit to the target, moved on to search elsewhere before eventually returning to the target and responding. This ‘target re-fixation pattern’ indicates that the target item was, on first inspection, mis-identified as a distractor, causing the participant to move away and search elsewhere, before eventually returning to the target. And the increased rate of such mis-identifications in the rare region in ASD is indicative of a bias towards identifying items at a previous distractor location as a distractor rather than a target when the appearance of a distractor at that location was surprising. This is in line with precision-regulation accounts, which assume an over-reliance on prediction errors in ASD, the consequence being that “every minor violation will induce new learning” (Van de Cruys et al. 2014, p. 653). When a distractor unexpectedly appeared at a rare location, the large prediction error or ‘surprise’ produced by the distractor triggered new learning, giving rise to a short-lived conservative prior belief that items at that location are likely to be distractors. Distractors appearing in the frequent region, on the other hand, caused less surprise, and therefore would not, or to a lesser extent, trigger new learning (except at the very beginning of the experiment) because participants quickly learnt to expect that distractors would frequently appear there. The reaction to rare distractor events is consistent with Falter et al.’s (2012) finding of a more conservative response bias in a time perception task in ASD, which might thus reflect a general characteristic of the autistic cognitive style, serving the purpose of regulating perceived environmental volatility.

However, while being consistent with a precision-regulation account, this leaves open the question of how the development of such a short-term prior is related to the learning of the long-term prior, the latter being responsible for the distractor-location probability-cueing effect. That is, why do ASD individuals acquire as ‘good’ a long-term prior for the underlying spatial distractor distribution as TD individuals reducing the attention-capturing power of distractors in the frequent versus the rare distractor region, when they actually overreact to distractor events in the rare region, adopting a strong belief that a future items at the location of an established distractor in this region is likely also a distractor, rather than a target? Should this over-reaction not lead to a strengthening of spatial distractor suppression in the rare region, at the expense of the frequent region, resulting in a ‘flatter’ prior compared to TD individuals?

**Fig. 7:**
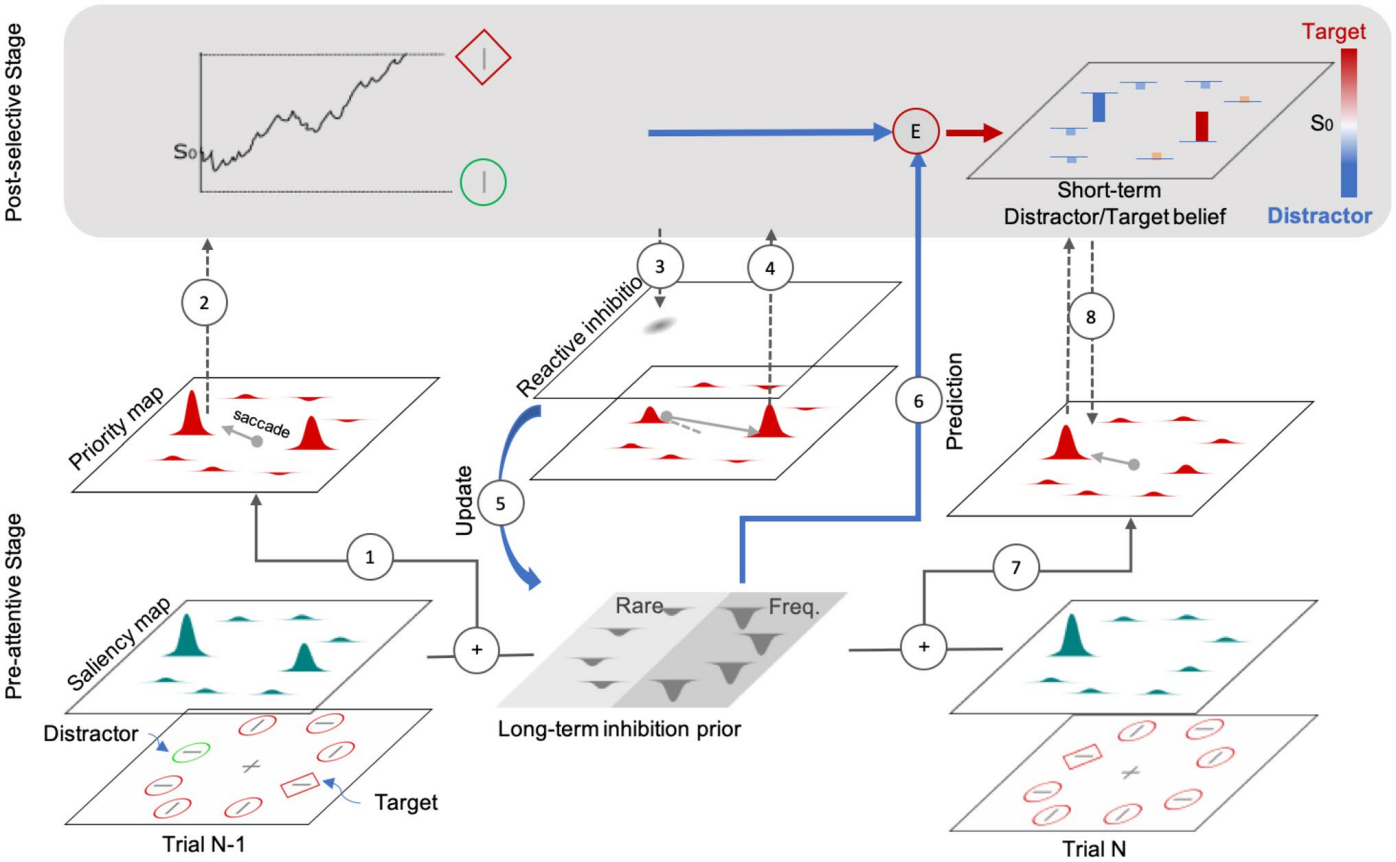
Illustration of the interaction between pre-attentive and post-selective processing and the updating of the associated long- and short-term priors. In the depicted trial *N* − 1, the highly salient singleton distractor (see saliency map) in the rare distractor region generates a strong attentional-priority signal (*1*), capturing the first saccade and bringing the distractor into the focus of attention. After identifying it as a distractor (*2*), reactive inhibition (*3*) is applied to its location on the priority map, reducing the distractor peak below the peak of the target. This triggers the second saccade to the next most salient item, the target, which is then identified as such and a manual response is issued (*4*). Applying reactive inhibition to a distractor location updates the long-term prior of the distractor-frequency map (*5*), which regulates the amount of proactive inhibition applied to the various locations in the frequent and rare regions (essentially a location-specific scaling factor applied to the saliency signals in computing attentional priority). At the post-selective stage, identifying the item fixated first as a distractor is compared to the likelihood of a distractor occurring at this location (*6*), which is represented by the map of the long-term inhibition prior; in the example, this likelihood was low as the distractor occurred in the rare region. This yields a prediction error (‘surprise’), which is used to adjust the starting point (*S*_*0*_) of the evidence-accumulation process towards the distractor boundary; in the example, the prediction error was big, bringing about a large shift of *S*_*0*_ towards the ‘distractor’ boundary. That is, the *S*_*0*_ values are dynamically updated and buffered in a short-term ‘target/distractor belief’ map, which then influences the post-selective decisions on the next trial. ASD individuals assign a greater weight to the prediction error, translating into a larger shift of *S*_*0*_ towards a ‘distractor’ decision. When, on the next trial, a target appears at the previous distractor location (in the rare region), it produces a strong priority signal (proactive inhibition is weak for locations in the rare region) and, thus, attracts the eye immediately (*7*). However, given a noisy (‘jittery’) evidence accumulation process, the shift of *S*_*0*_ towards the distractor boundary (carried over from the previous trial) will increase the chance of incorrect ‘distractor’ decisions (i.e., the accumulated evidence reaching the ‘distractor’, rather than the ‘target’, boundary) (*8*). This leads to an extended search process (involving the scanning of other, non-target locations) until the target is eventually detected

The answer would depend on how the two, pre- and post-selective levels communicate. The scenario we envisage, based on prior considerations (Sauter et al. 2020), is illustrated in Fig. 7. Plausibly, the long-term prior about the spatial distractor distribution is indeed acquired from observers’ explicit experience of distractor events: the most salient item in the display captures attention, and the eye, and is explicitly identified and ‘rejected’ as a task-irrelevant distractor. However, to find the target, identification of an item as a distractor at the post-selective stage would have to be followed by inhibition of its priority signal (its peak) on the search-guiding priority map, so that attention can be disengaged from the distractor and re-oriented to the target, whose location is ‘flagged’ by the second highest peak on the priority map (see Fig. 7). This process of reactive positional inhibition of ‘rejected’ locations—conceived of as an IOR-type process (e.g., Koch and Ullman 1985)—is likely to provide the ‘(error) signal’ for the learning of the pre-selective (inhibition) prior. Given that more distractors occur in the frequent region, more IOR-type ‘rejection’ signals are generated in this region, biasing pro-active, tonic inhibition at the pre-selective stage towards the likely distractor region. On this account, the amount of reactive inhibition required to release attention from a distractor that captured attention and the eye depends on the relative strength of the distractor versus the target priority signals, not on the strength of the prediction error. The latter, however, is used to update the post-selective distractor/target ‘belief’, that is, the expected likelihood of a distractor-vs.-target event at the post-selective decision stage. In our paradigm, the likelihood that a selected item is a target is equal for all display locations; however, while the likelihood that the selected item is a distractor is similarly high for locations in the frequent distractor region, it is much lower than the target likelihood for locations in the rare region. Thus, when a distractor in the rare region captures attention, it creates a large prediction error, and this leads to an adjustment of the short-term post-selective target/distractor belief towards a ‘distractor’ decision (Fig. 7 right-upper panel for an illustration). Individuals with ASD assign a greater weight to this prediction error, so the result is an overadjustment compared to TD individuals. However, in ASD, only the strength of this post-selective belief is increased for distractors in the rare region, but not the strength of the priority signals they generate. The latter determines the likelihood that such a distractor captures attention, which was not higher in ASD compared to TD individuals. Accordingly, there would be no reason why individuals with ASD would differ from TD individuals in terms of the long-term inhibition prior they develop.

According to recent theoretical considerations, the aberrant social-cognitive development in ASD arises, at least in part, from attentional atypicalities, such as in attentional re-orienting (Keehn et al. 2013, 2016; Orekhova and Stroganova 2014; Spaniol et al. 2018). Yet, the current results show entirely intact low-level mechanisms of attentional-priority regulation in individuals with ASD, and what, on the behavioral level, appears like a re-orienting deficit (slowed responses to ‘coincident’ targets) rather reflects altered post-selective decision processes, in particular, over-adjustment of decision parameters upon encountering surprising stimuli. Thus, speculatively, individuals with ASD control surprise not just in complex scenarios where surprise avoidance goes along with behavioral rigidity; rather, mirroring these higher-level processes, they attempt to regulate surprise down to the level of simple perceptual decision-making.

The results of the current study need to be interpreted in the light of our sample consisting of high-functioning adults with ASD, limiting generalisability to the whole spectrum. The reason for this limitation is the ethical consideration that new paradigms should first be tested with participants who can consent for themselves. Our findings can now form the basis for future studies testing replicability in children with ASD and individuals with comorbid learning disability.

We conclude that individuals with ASD can, to an equal degree as TD individuals, make use of prior information about where an irrelevant distractor is most likely to appear when deciding where to direct attention. However, when a distractor appears at an unlikely, ‘surprising’ location, individuals with ASD are more likely to overreact by coming to expect future items occurring at that position to be distractors, which makes them prone to fail to correctly identify targets appearing at that location. This pattern could reflect a general tendency of individuals with ASD to assign atypically high weight to perceptual prediction errors, even when such prediction errors cannot be eliminated by further learning and so should be ignored as irrelevant noise, as has been suggested by some predictive-coding theories of autism.

There is a consensus that understanding the sensory abnormalities in ASD—such as why people with ASD can be overly sensitive to sensory input—is of paramount theoretical importance (Autistica 2016), and the predictive-coding framework has high potential in explaining this perceptual and cognitive aspect of ASD. The current findings add towards a refinement of this approach by suggesting that overrating of prediction errors and undue adjustment of prior beliefs, especially in response to unexpected events, might contribute to the sensory oversensitivity that is characteristic of ASD.

## Supplementary Information

Below is the link to the electronic supplementary material.

Supplementary file1 (DOCX 2085 KB)

## Footnotes

1 In more detail, Lawson et al. (2017) devised a hierarchical Gaussian filter, which performed Bayesian updating of a probabilistic model with three levels: the lowest level represented a prediction of the outcome on a particular trial, the second level the conditional probabilities of the different outcomes, and the third the volatility of the environment (i.e., the rate at which the outcome probabilities change).

2 For this analysis, we excluded trials on which the target position on trial *n* coincided with the distractor position on trial *n* – 1. Also, two participants from each group had to be excluded because there were no trials for the 2-back coincidence condition in the rare region after excluding error trials and 1-back coincidence trials.

3 On distractor-absent trials, by contrast, the proportion of first saccades to the target was unaffected by the target location; but the saccadic latencies were slower to targets appearing in the frequent vs. rare distractor region, for both ASD and TD individuals. See Supplementary for details.

4 In both groups, compared to non-coincident trials, the onset of the first saccade to the target was numerically delayed, by some 40 ms, when the target appeared at the previous distractor location in the rare region [*t*(30) = 1.96, *p* = 0.06], consistent with reactive cross-trial IOR of the preceding distractor location; the strength of this effect was comparable between ASD and TD individuals.
