# Supplementary material for: Acquisition and Use of ‘Priors’ in Autism: Typical in Deciding Where to Look, Atypical in Deciding What Is There
Source: J Autism Dev Disord. 2020 Dec 29;51(10):3744–58. doi: 10.1007/s10803-020-04828-2 (PMC8460564; doi:10.1007/s10803-020-04828-2)
Supplement: Supplementary file 1 — Supplementary file1 (DOCX 2085 KB) [file 10803_2020_4828_MOESM1_ESM.docx]

# **Supplementary**

# **Acquisition and Use of ‘Priors’ in Autism:**

# **Typical in Deciding Where to Look, Atypical in Deciding What is There**

Fredrik Allenmark^1*^, Zhuanghua Shi^1^, Rasmus L. Pistorius^2^, Laura A. Theisinger^2^,

Nikolaos Koutsouleris^2^, Peter Falkai^2^,

Hermann J. Müller^1^ & Christine Falter-Wagner^2*^

1. Department of Psychology, Ludwig-Maximilians-Universität München, Munich, Germany
2. Department of Psychiatry and Psychotherapy, Ludwig-Maximilians-Universität München, Munich, Germany

***Eye-movement analysis: Method and Results***

**Method**

Movements of participants’ dominant eye were monitored using an Eyelink 1000 desktop-mounted system (SR Research, Canada), set at a sampling rate of 1 kHz. Stimulus presentation, response recording, and eye movement sampling were controlled via a customized Matlab program using the Psychtoolbox and the Eyelink Toolbox [(Brainard, 1997)](https://paperpile.com/c/FufjZj/JF072).

Given that participants were told to fixate the (central) fixation cross at the start of each trial until the appearance of the search display, the fixation before display onset was assumed to be centered on the central marker. Consequently, any divergence of the position reported by the eye-tracker for this fixation and the position of the fixation cross was considered due a calibration error, and the positions of all other fixations during that trial were corrected by subtracting this error. Next, for examining the oculomotor scanning pattern through the trial display, a fixation was counted as a fixation on a particular item in the search display if it was closer to that item than to any other item or the fixation cross. Fixations that were closer to the fixation cross than to any item in the search display were discounted for analyzing which item was fixated first.

Five of the participants, two in the ASD group and three in the TD group, were not included in the eye-movement analyses, because we were unable to obtain adequate eye-movement data for these participants. Additionally, more participants were excluded from some of the analyses, because of a lack of trials in particular conditions, as described in more detail below.

**Results**

*Distractor-location effects (distractor present trials):*

[Figure S](https://docs.google.com/document/d/1Rs8_SwHc4FQv6VXoM8MitkzH7BXgJsOHtuJQr4C-AUc/edit#bookmark=id.2et92p0)1 presents the average number of fixations made between the onset of the search display and the response on distractor-present trials, dependent on the distractor condition (distractor in rare vs. frequent region). Significantly fewer fixations were made on trials on which the distractor appeared in the frequent versus the rare region (F(1, 37) = 10.11, p < .01). Numerically the difference was smaller in the ASD compared to TD group, but the interaction was not statistically significant (F(1, 37) = 1.96, p = 0.17).


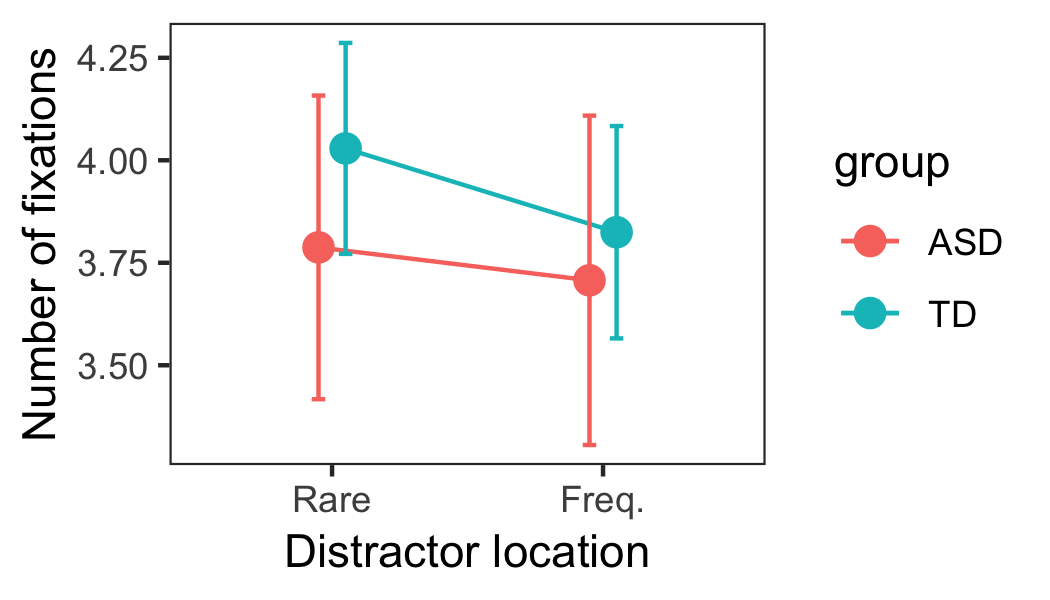


[*Figure S*](https://docs.google.com/document/d/1Rs8_SwHc4FQv6VXoM8MitkzH7BXgJsOHtuJQr4C-AUc/edit#bookmark=id.tyjcwt)*1:* Average number of fixations between search display onset and response on distractor-present trials, dependent on the distractor location (in rare vs. frequent distractor region).

[Figure S](https://docs.google.com/document/d/1Rs8_SwHc4FQv6VXoM8MitkzH7BXgJsOHtuJQr4C-AUc/edit#bookmark=id.3dy6vkm)2 presents the proportions of (distractor-present) trials on which the first fixated item in the search display was the target (left panel) or, respectively, the distractor (right panel) trials. A larger proportion of first fixations fell on the target and a smaller proportion on the distractor when the distractor appeared in the frequent compared to the rare region (target: F(1, 37) = 38.01, p < .001; distractor: F(1, 37) = 9.90, p < .01). Neither effect differed significantly between groups.


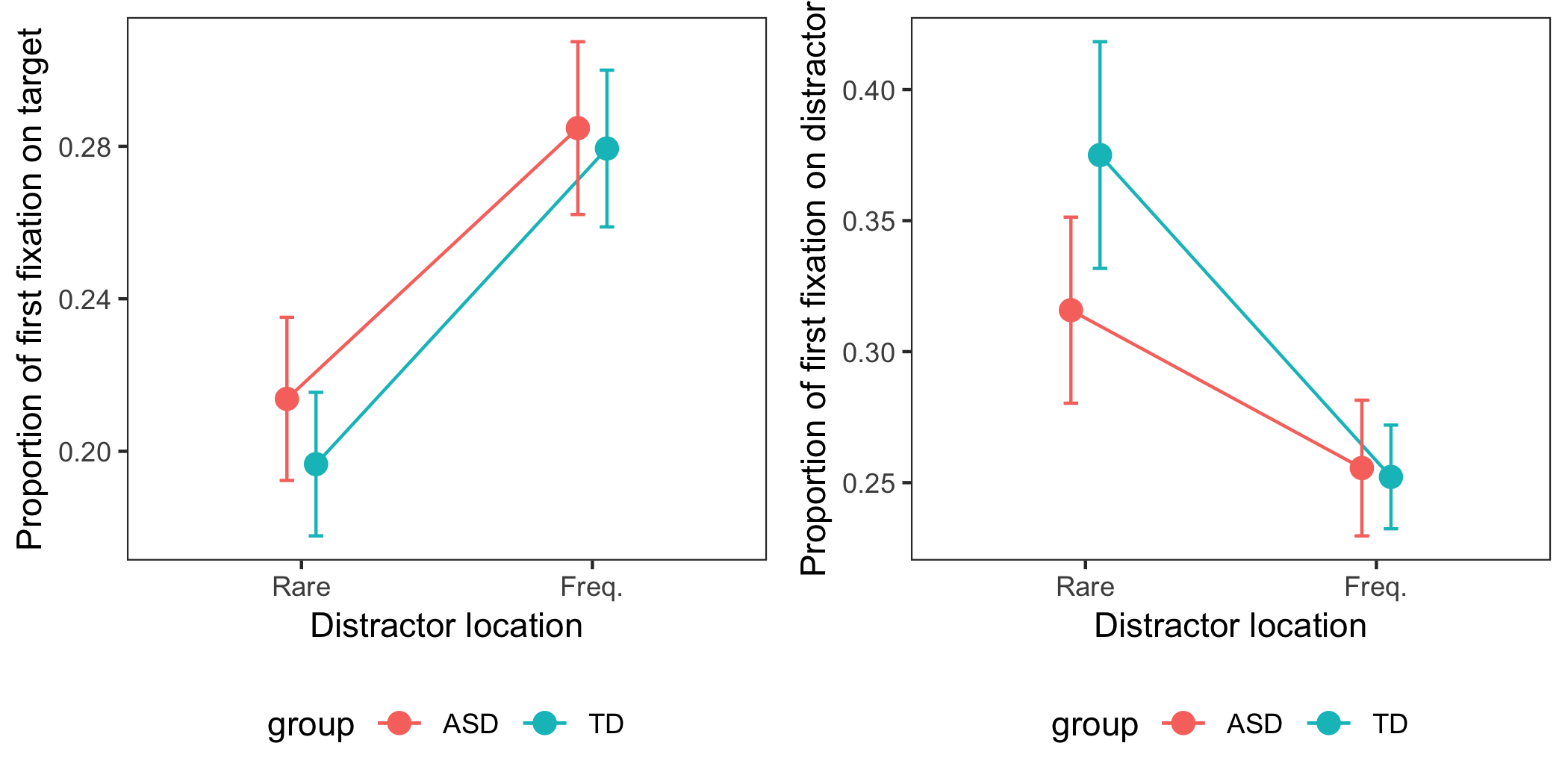


[*Figure S*](https://docs.google.com/document/d/1Rs8_SwHc4FQv6VXoM8MitkzH7BXgJsOHtuJQr4C-AUc/edit#bookmark=id.1t3h5sf)*2:* Proportion of first fixations on the target (left) and, respectively, on the distractor (right), dependent on the distractor location (in rare vs. frequent distractor region).

[Figure S](https://docs.google.com/document/d/1Rs8_SwHc4FQv6VXoM8MitkzH7BXgJsOHtuJQr4C-AUc/edit#bookmark=id.4d34og8)3 presents the average of individual participants’ median durations of the first fixation on the distractor. This analysis only includes trials on which there was at least one fixation on the distractor. [One participant in the control group had to be excluded because there were no fixations on distractors in the rare region; this participant made few eye movements away from fixation overall, indicative of a search strategy largely relying on covert attention shifts.]

The first distractor fixations were marginally shorter for distractors in the frequent compared to the rare region (F(1, 36) = 3.82, p = 0.06). Although the difference appeared numerically smaller in the ASD, compared to the TD, group, the interaction with group was not significant (F(1, 36) = 1.40, p = 0.24).


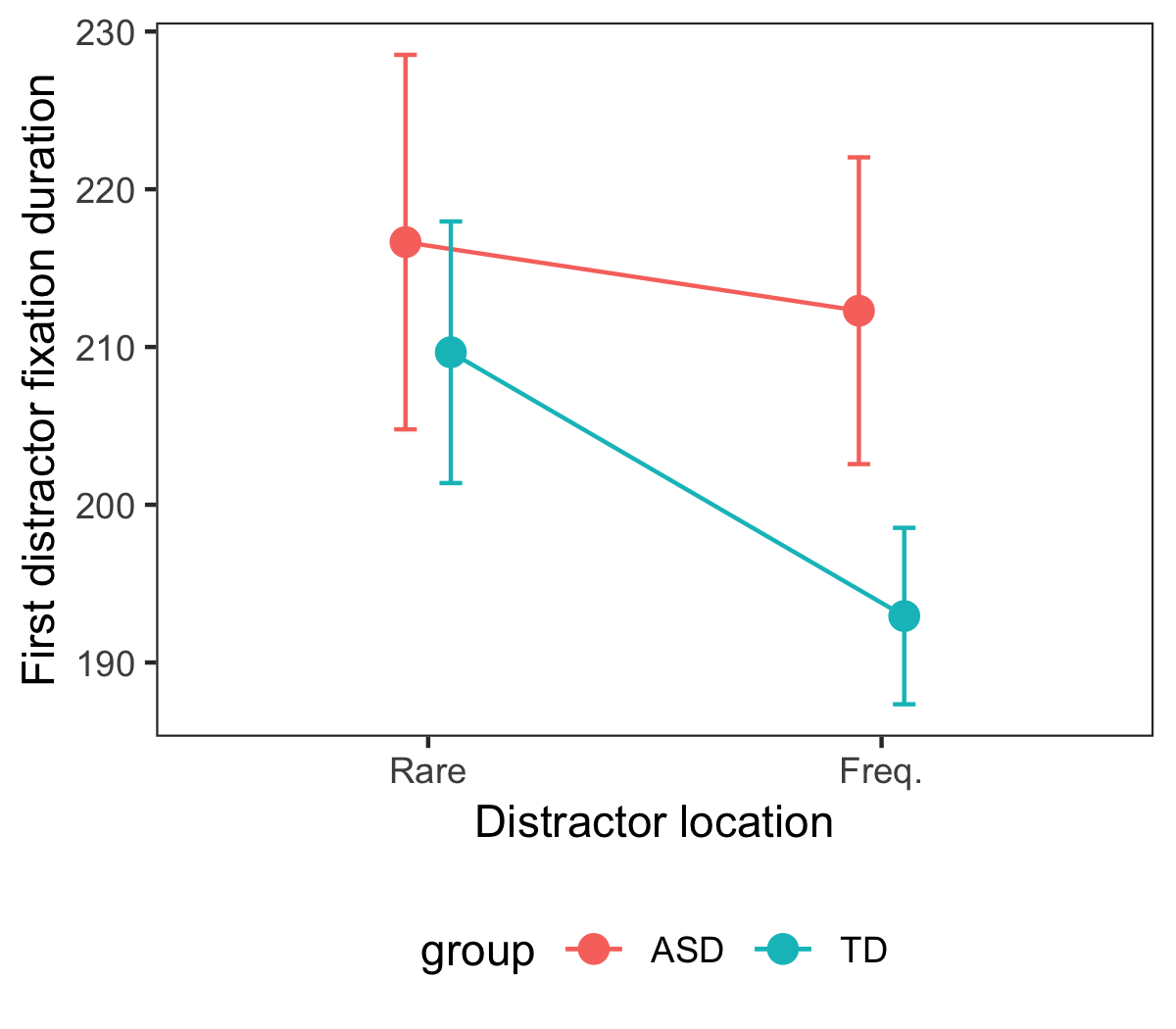


[*Figure S*](https://docs.google.com/document/d/1Rs8_SwHc4FQv6VXoM8MitkzH7BXgJsOHtuJQr4C-AUc/edit#bookmark=id.2s8eyo1)*3:* Average median duration of the first distractor fixation.

*Target-location effect (distractor-absent trials):*

[Figure S](https://docs.google.com/document/d/1Rs8_SwHc4FQv6VXoM8MitkzH7BXgJsOHtuJQr4C-AUc/edit#bookmark=id.17dp8vu)4 presents the average number of fixations between the onset of the search display and the response on distractor-absent trials. There was no significant difference in the number of fixations depending on whether the target appeared in the frequent or the rare distractor region (F(1,37) = 2.24, p=0.14).


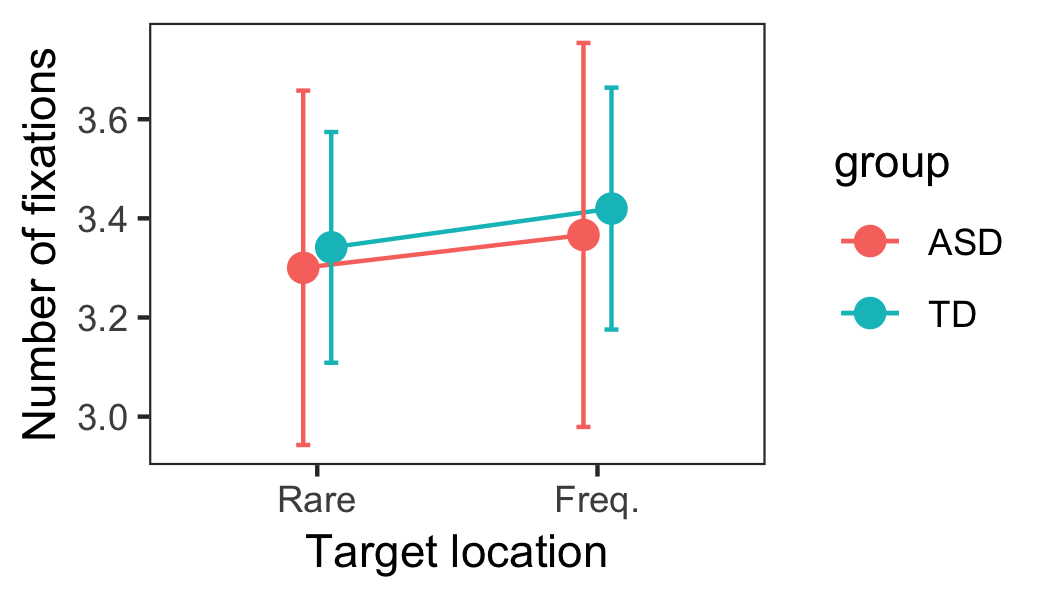


[*Figure S*](https://docs.google.com/document/d/1Rs8_SwHc4FQv6VXoM8MitkzH7BXgJsOHtuJQr4C-AUc/edit#bookmark=id.3rdcrjn)*4:* Average number of fixations between search display onset and response on distractor-absent trials, dependent on the target location (in rare vs. frequent distractor region).

[Figure S](https://docs.google.com/document/d/1Rs8_SwHc4FQv6VXoM8MitkzH7BXgJsOHtuJQr4C-AUc/edit#bookmark=id.26in1rg)5 shows the proportions of (distractor-absent) trials on which the first fixated item in the search display was the target, dependent on the target location (target at rare vs. frequent distractor location) (left panel); and the average (across participants) of the median saccadic onset latencies on trials on which the target was fixated first (right panel). For the proportion of first target fixations, neither the main effect of target condition nor the interaction with group approached significance (both F < 0.2). However, the onset latencies of saccades to the target were generally slower when the target appeared in the frequent versus the rare distractor region (F(1,37) = 11.27, p < 0.01; the interaction with group was not significant (F(1,37) = 1.62, p = 0.21).


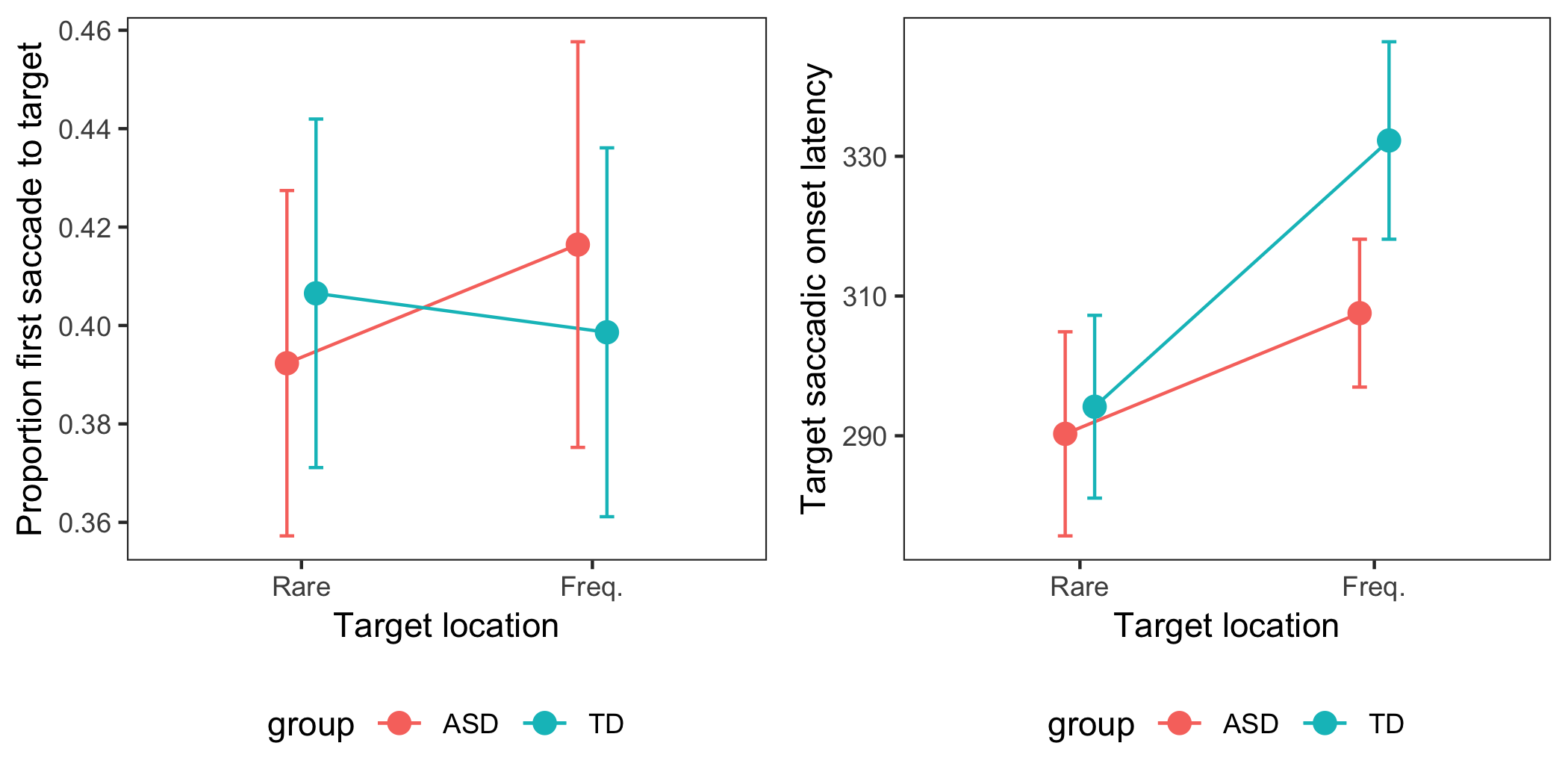


[*Figure S*](https://docs.google.com/document/d/1Rs8_SwHc4FQv6VXoM8MitkzH7BXgJsOHtuJQr4C-AUc/edit#bookmark=id.lnxbz9)*5:* Proportion of first fixations on the target on distractor-absent trials (left), and average median onset latency of the first saccades on trials on which the target was fixated first (right).

*Distractor_n-1_–target_n_ (spatial) coincidence effects:*

[Figure S](https://docs.google.com/document/d/1Rs8_SwHc4FQv6VXoM8MitkzH7BXgJsOHtuJQr4C-AUc/edit#bookmark=id.35nkun2)6 shows the effect of the spatial coincidence of the distractor position on trial *n-1* and the target position on trial *n* on the proportion of first fixations on the target, dependent on the target location on trial *n* (in frequent vs. rare distractor region). Numerically, ASD participants fixated the target more often before any other item on coincident compared to non-coincident trials when the target appeared at one of the rare distractor locations (marginally significant effect: t(19) = 2.07, p = 0.052).


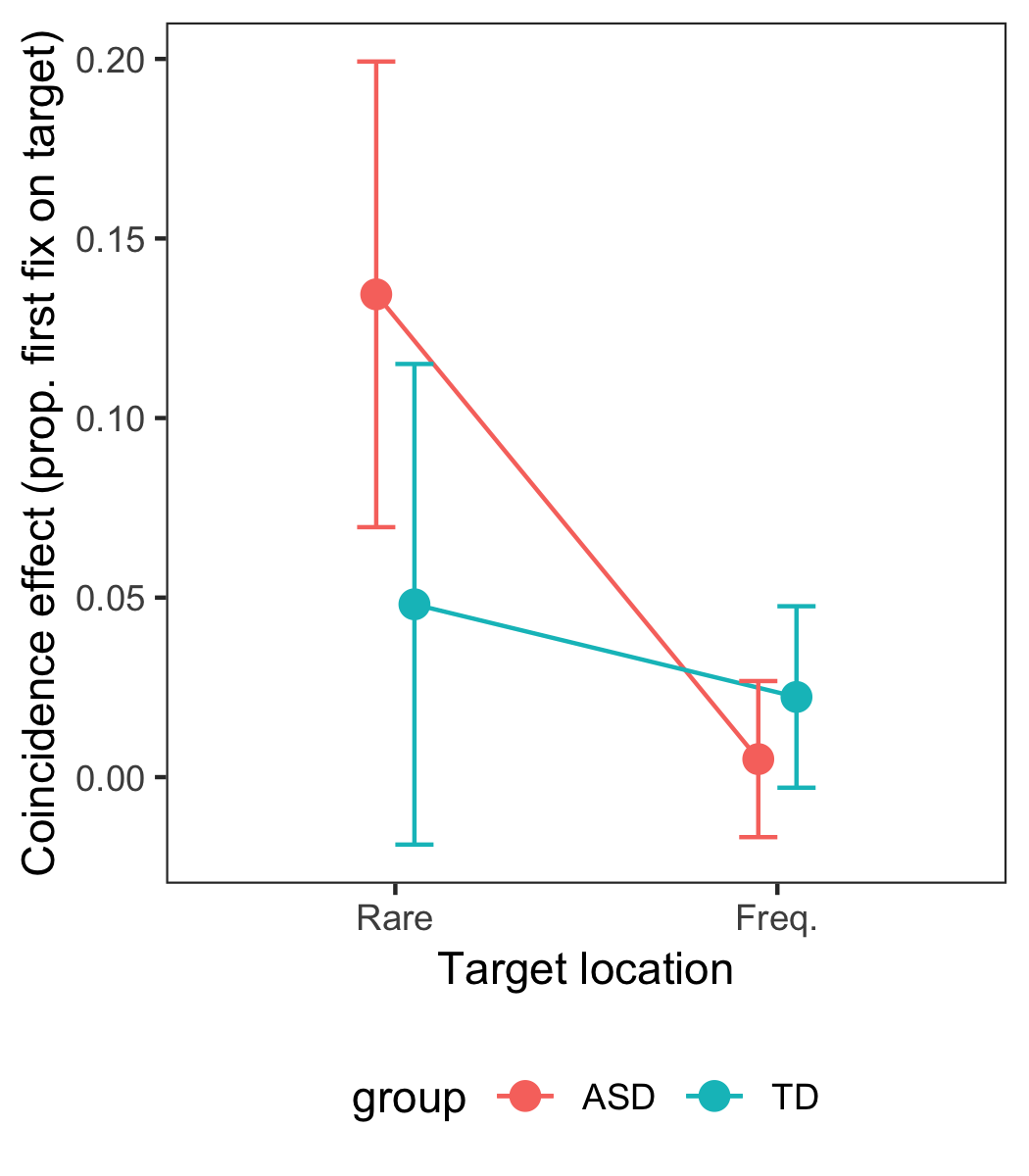


[*Figure S*](https://docs.google.com/document/d/1Rs8_SwHc4FQv6VXoM8MitkzH7BXgJsOHtuJQr4C-AUc/edit#bookmark=id.1ksv4uv)6: Effect of spatial coincidence (of the distractor position on trial *n-1* and the target position on trial *n*) on the proportion of first fixations on the target, i.e. the difference between this proportion on coincident trials compared to non-coincident trials, dependent on the target location (in rare vs. frequent distractor region).

[Figure S](https://docs.google.com/document/d/1Rs8_SwHc4FQv6VXoM8MitkzH7BXgJsOHtuJQr4C-AUc/edit#bookmark=id.44sinio)7 presents the average median onset latencies of the first saccade on those trials on which the target was fixated first. [For this analysis, four participants in each group had to be excluded because they had no ‘valid’ trials in one of the conditions.] In both groups, the onset of the first saccade to the target was numerically delayed, by some 40 ms, when the target appeared at the previous distractor location in the rare region (though this – target condition x intertrial interaction – effect was only marginally significant: F(1, 29) = 3.59, p = 0.068).


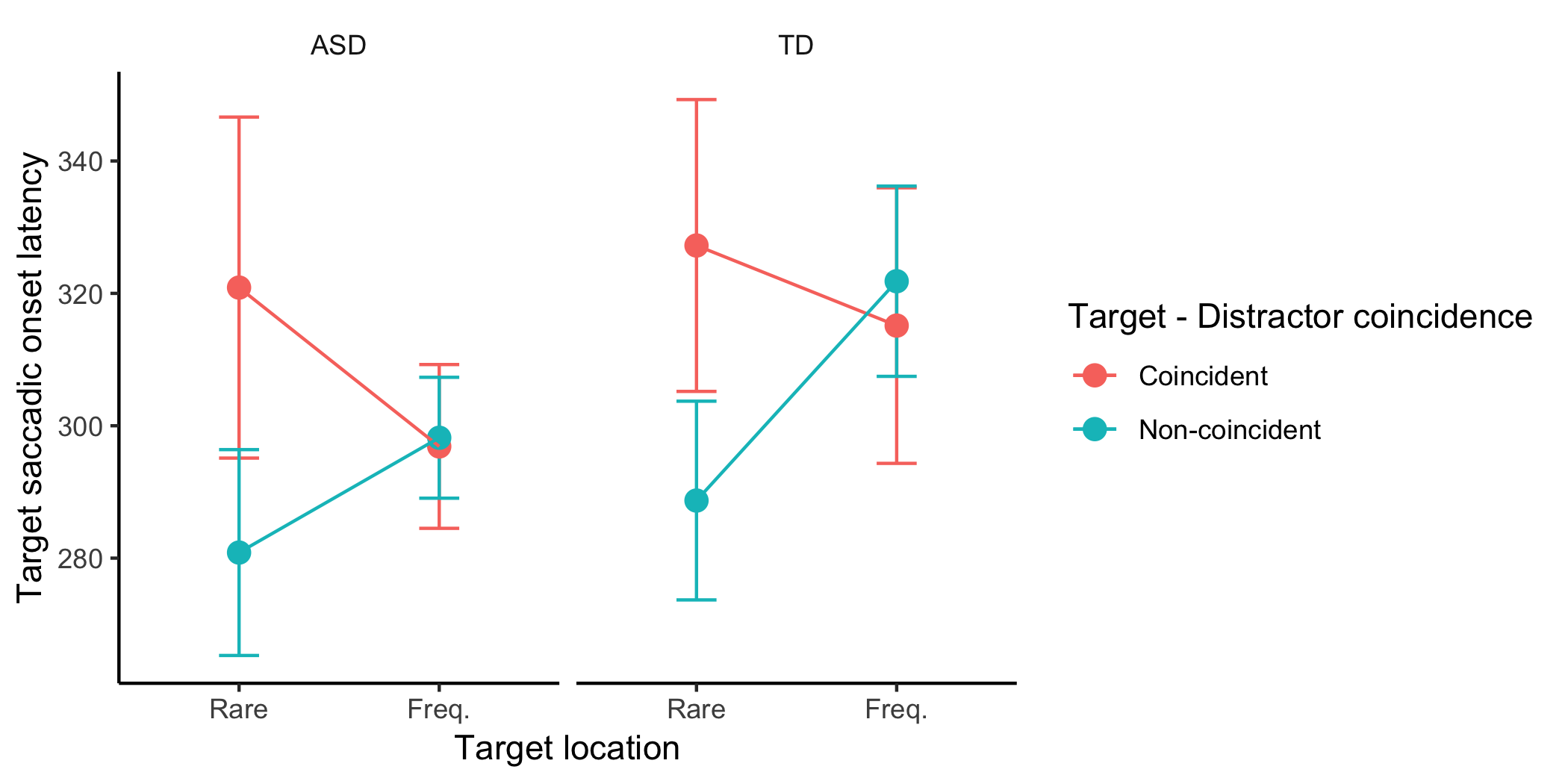


[*Figure S*](https://docs.google.com/document/d/1Rs8_SwHc4FQv6VXoM8MitkzH7BXgJsOHtuJQr4C-AUc/edit#bookmark=id.2jxsxqh)7: Average median onset latencies of the first saccades on trials on which the target was fixated first, for targets appearing in the frequent and rare distractor region, separately for trials on which the target appeared at the previous distractor location (coincident) and, respectively, a non-distractor location (non-coincident).

[Figure S](https://docs.google.com/document/d/1Rs8_SwHc4FQv6VXoM8MitkzH7BXgJsOHtuJQr4C-AUc/edit#bookmark=id.z337ya)8 presents the effect of spatial coincidence (of the distractor location on trial *n-1* and the target location on trial *n*) on the average number of fixations per trial, dependent on the target location (at frequent vs. rare distractor location). This ‘coincidence effect’ reflects *the number of extra fixations made on trials on which the target appeared at a previous distractor (vs. a non-distractor) location*. [For this analysis, two participants in the TD group had to be excluded because there were no remaining coincident trials for targets in the rare distractor region after excluding error trials and trials without fixations after search display onset.] Numerically, ASD participants made an increased number of fixations on coincident, as compared to non-coincident, trials when the target appeared at one of the rare distractor locations (marginally significant effect: t(19) = 1.89, p = 0.07). However, an ANOVA on the coincidence effect on number of fixations with group (ASD or TD) and target position (frequent or rare region) as factors revealed that neither the effect of group (F(1, 35) = 3.0, p = 0.09), the effect of target location (F(1, 35) = 2.1, p = 0.15) nor the interaction (F(1, 35) = 1.7, p = 0.2) was significant.


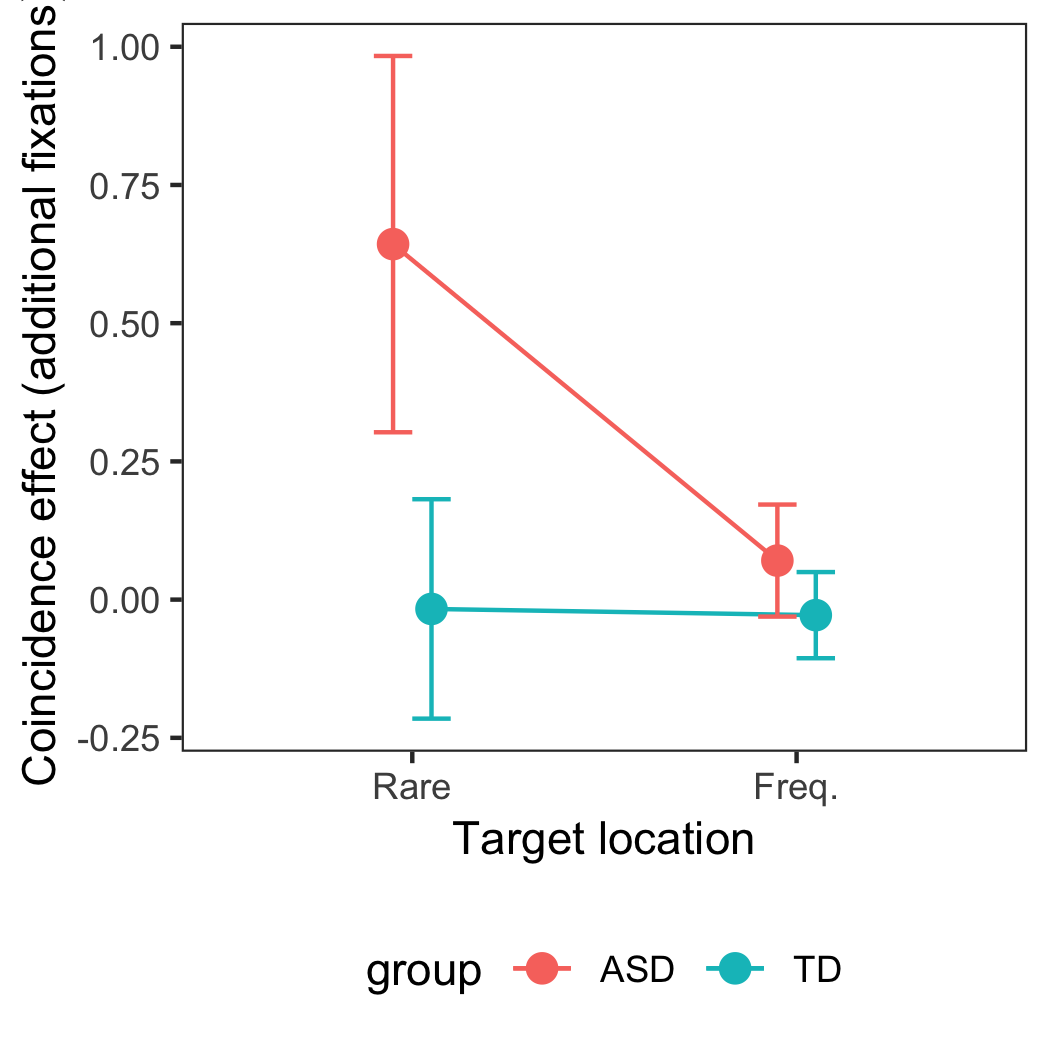


[*Figure S*](https://docs.google.com/document/d/1Rs8_SwHc4FQv6VXoM8MitkzH7BXgJsOHtuJQr4C-AUc/edit#bookmark=id.3j2qqm3)*8:* Effect of spatial coincidence (of the distractor position on trial *n-1* and the target position on trial *n*) on the average number of fixations per trial, dependent on the target location on trial *n* (in rare vs. frequent distractor region). What is depicted is the number of extra fixations made on coincident (minus non-coincident) trials.

*Target-fixation patterns on coincident trials:*

To better understand how ASD individuals’ oculomotor search patterns differed from those of TD individuals when target *n* appeared at the location of the previous, *n-1* distractor in the rare region, we split the (distractor-absent) data into different categories based on the ‘target fixation pattern’. This was done after excluding all trials on which there were no confirmed target fixations; also, for each category, multiple fixations in a row on the target were treated as a single target fixation and their durations were added together, providing a measure of the ‘target dwell time’. Category 1 consisted of trials on which the target was fixated a single time (followed by at most one fixation elsewhere) before response (henceforth referred to as ‘*single final target fixation*’ trials); category 2 consisted of trials on which the target was fixated a single time, followed by multiple fixations on other items before response (‘*single non-final target fixation*’ trials); and category 3 consisted of trials on which the target was fixated more than once with at least one non-target fixation in between (‘*target re-fixation*’ trials).

Median RTs on distractor-absent trials differed significantly between the different target-fixation pattern conditions (see [Figure S](https://docs.google.com/document/d/1Rs8_SwHc4FQv6VXoM8MitkzH7BXgJsOHtuJQr4C-AUc/edit#bookmark=id.1y810tw)9; F(2,62) = 123.9, p < .001): RTs were overall slowest in the ‘target re-fixation’ condition and fastest in the ‘single final target fixation’ condition. There was no significant interaction with group (ASD vs. TD) or target condition (target located in frequent vs. rare region). [Three participants in each group had to be excluded from this analysis because there were no trials in one of the conditions.]


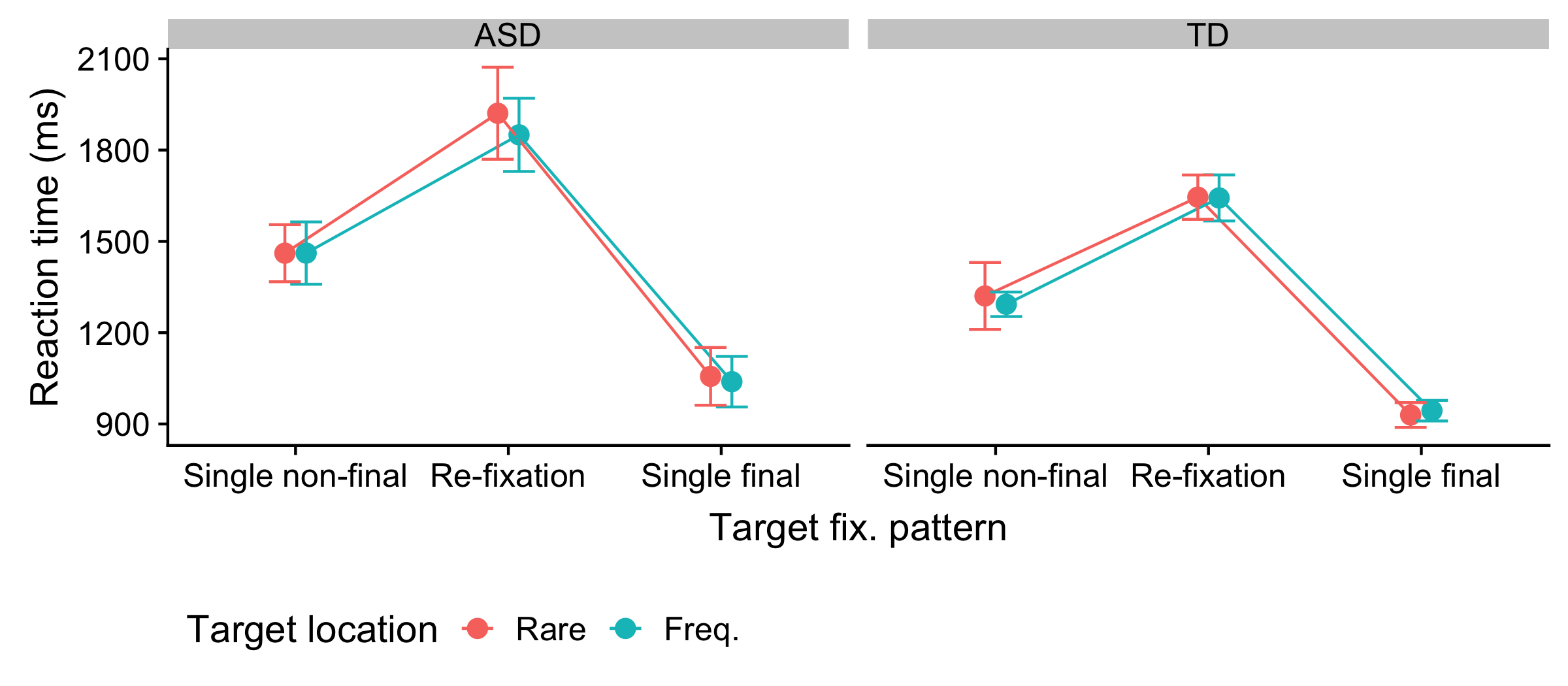


[*Figure S*](https://docs.google.com/document/d/1Rs8_SwHc4FQv6VXoM8MitkzH7BXgJsOHtuJQr4C-AUc/edit#bookmark=id.4i7ojhp)*9:* Average median RTs on distractor-absent trials for the different target fixation patterns, separately for ASD and TD individuals, depending on the target location (in rare vs. frequent distractor region).

[Figure S1](https://docs.google.com/document/d/1Rs8_SwHc4FQv6VXoM8MitkzH7BXgJsOHtuJQr4C-AUc/edit#bookmark=id.2xcytpi)0 presents the dwell times of the first ‘fixation’ on the target for the three target-fixation patterns. These times differed significantly among the three conditions (F(2, 62) = 23.0, p < .001). In particular, the dwell times on the target were longest in the ‘single final’ condition (compared to either of the other conditions), for which the RTs had been shortest (see Figure S10). This pattern was not significantly influenced by the target location (in the frequent vs. the rare distractor region), or the participant group (ASD vs. TD individuals). [Three participants in each group had to be excluded from this analysis because there were no trials in one of the conditions.]


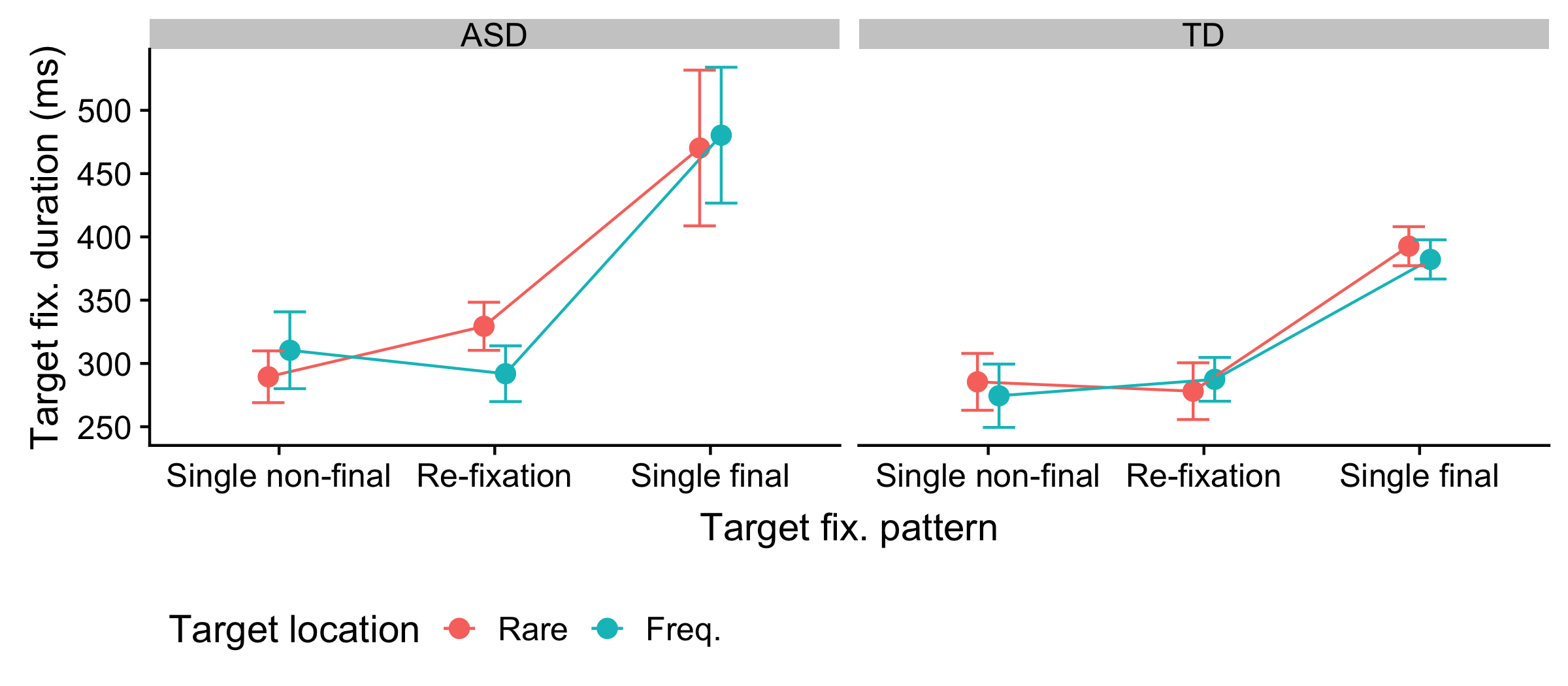


[*Figure S1*](https://docs.google.com/document/d/1Rs8_SwHc4FQv6VXoM8MitkzH7BXgJsOHtuJQr4C-AUc/edit#bookmark=id.1ci93xb)*0*: Average median target fixation dwell time (first ‘fixation’) on distractor-absent trials for the different target fixation patterns, separately for ASD and TD individuals.

We next examined RTs dependent on the target fixation patterns in the critical condition in which ASD individuals exhibited the large coincidence effect, i.e.: distractor-absent trials on which a target appeared in the rare distractor region. For this, unlike the previous analyses, it was not possible to exclude participants with no trials in one or more condition, since almost every participant had missing trials in at least one condition. The following figure therefore depicts averages across different (but partially overlapping) sets of participants in the different conditions. [Figure S1](https://docs.google.com/document/d/1Rs8_SwHc4FQv6VXoM8MitkzH7BXgJsOHtuJQr4C-AUc/edit#bookmark=id.3whwml4)1 presents the RTs (distractor-absent trials with targets in the rare distractor region), dependent on whether the target appeared at the previous distractor location (coincident vs. non-coincident locations) and separately for the three target fixation patterns.

RTs do not differ much between coincident and non-coincident trials in either the ‘single final target fixation’ or the ‘target re-fixation’ conditions (Figure S12); they only differ in the ‘single non-final target fixation’ condition, where there is a cost in responding to coincident (vs. non-coincident) targets.


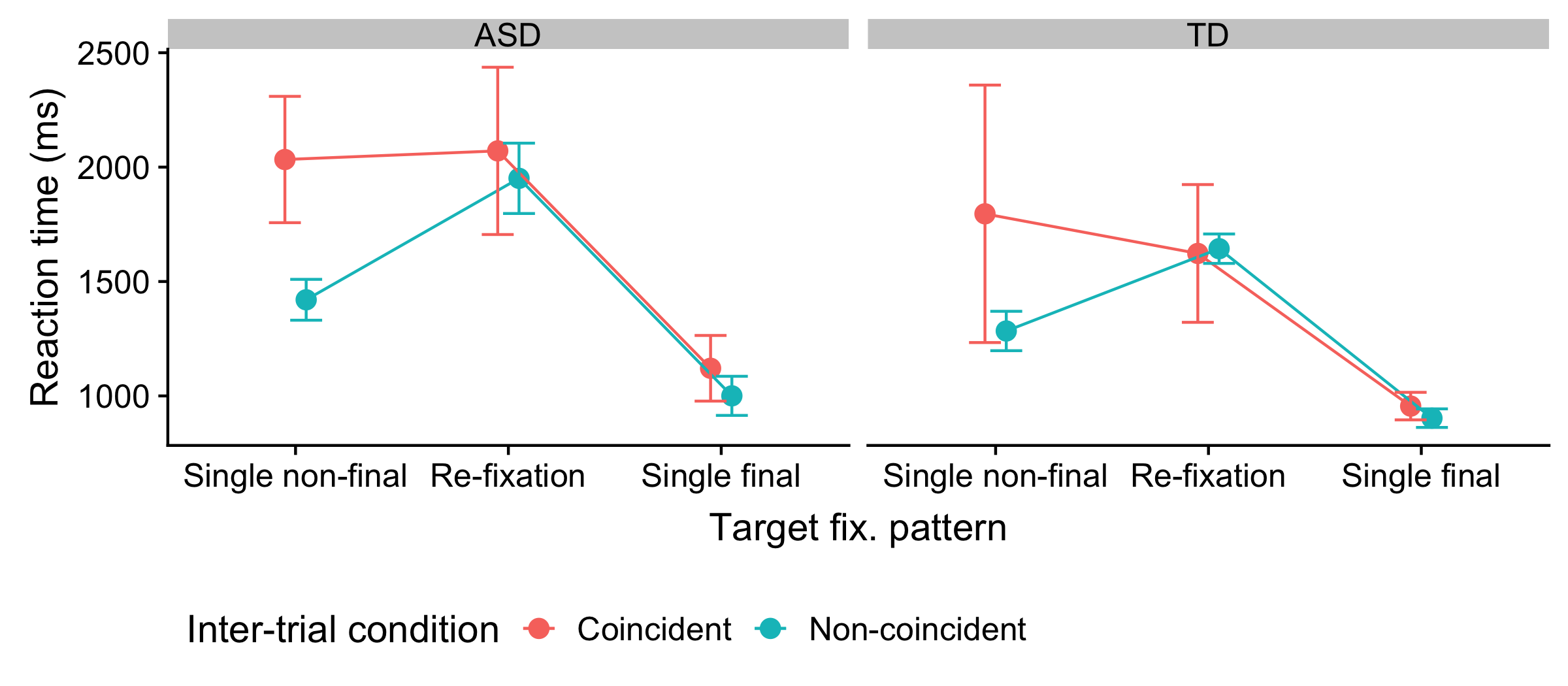


[*Figure S1*](https://docs.google.com/document/d/1Rs8_SwHc4FQv6VXoM8MitkzH7BXgJsOHtuJQr4C-AUc/edit#bookmark=id.qsh70q)*1*: RTs on distractor-absent trials on which the target appeared in the rare distractor region, for coincident (target appearing at previous distractor location) and non-coincident trials (target appearing at non-distractor location), separately for the different target fixation patterns.

Finally, we examined how the proportions of trials with the different target-fixation patterns (on distractor absent-trials on which the target appears in the rare distractor region) differ between the coincident and non-coincident conditions. The total number of trials with each fixation pattern in each condition was summed across participants in each group and divided by the total number of trials in each condition, also summed over participants in each group, in order to obtain the proportions of trials with each pattern. [Participants who had no trials in one of the conditions were excluded, resulting in the exclusion of four TD and three ASD participants.] [Figure S1](https://docs.google.com/document/d/1Rs8_SwHc4FQv6VXoM8MitkzH7BXgJsOHtuJQr4C-AUc/edit#bookmark=id.1pxezwc)2 depicts the resulting proportions. In the ASD group, there are fewer trials with the ‘single final’ pattern and more of the ‘single non-final’ and, in particular, “target re-fixation” patterns on the coincident versus non-coincident trials (on which the target did vs. did not appear at the previous distractor location). Chi-squared tests revealed the target fixation pattern distributions to differ significantly between coincident and non-coincident trials in the ASD group (𝛸^2^(2, N=1525) = 16.6, p < .001), but not in the TD group (𝛸^2^(2, N=1607) = 0.44, p = 0.80). On trials with a target in the frequent distractor region, the proportions were similar to proportions for non-coincident trials with a target in the rare distractor region and in particular, the proportion of the “target re-fixation” pattern was comparable (Coincident, ASD: 78% single final, 7% single non-final, 16% target refixation; Non-coincident, ASD: 80% single final, 7% single non-final, 13% target re-fixation; Coincident, TD: 70 % single final, 14% single non-final, 17% target re-fixation; Non-coincident, TD: 75% single final, 11% single non-final, 15% target-refixation) indicating that the high proportion of target re-fixations on coincident trials in the rare region is something unique to this particular condition (and to the ASD group).


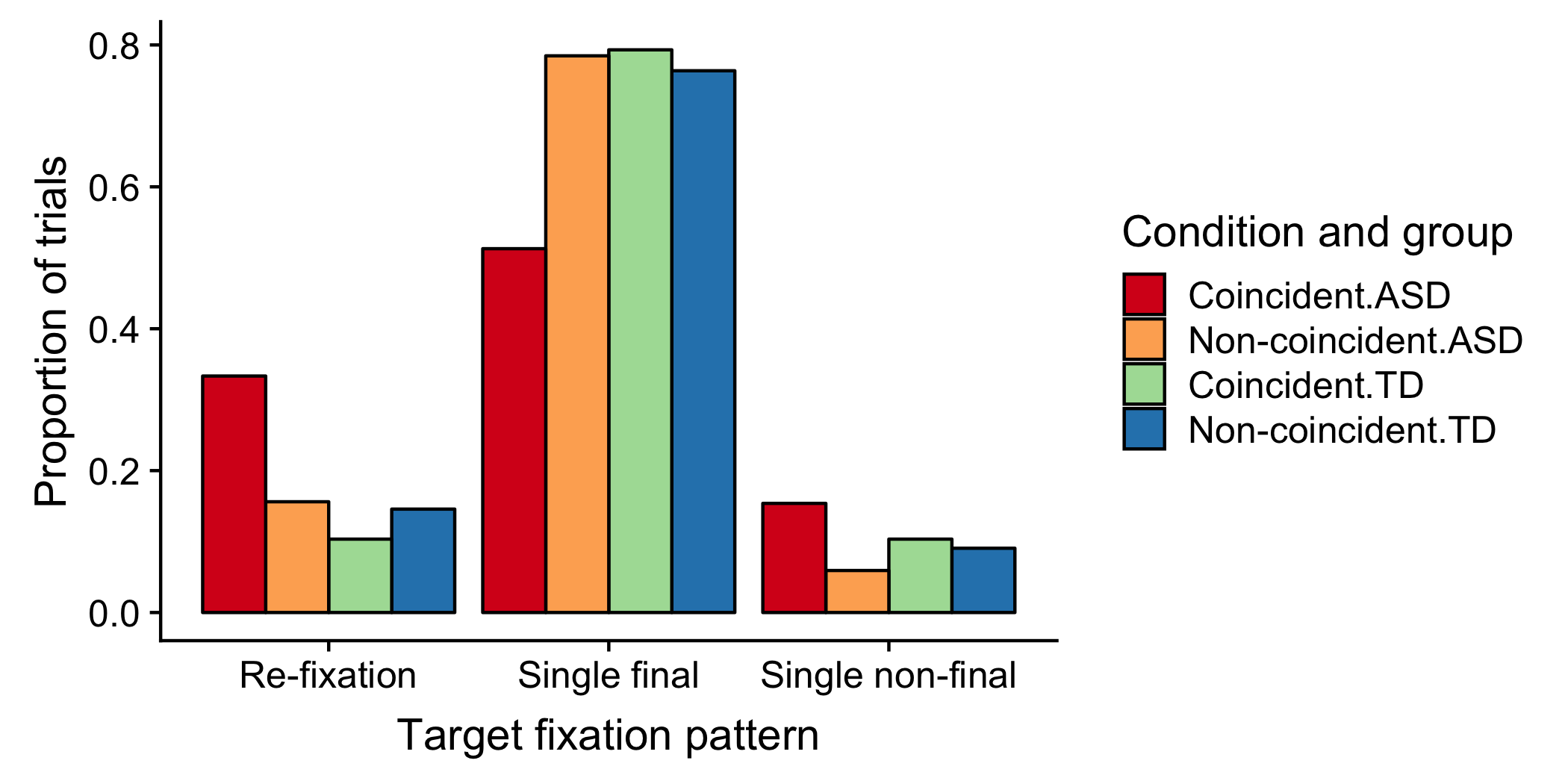


[*Figure S1*](https://docs.google.com/document/d/1Rs8_SwHc4FQv6VXoM8MitkzH7BXgJsOHtuJQr4C-AUc/edit#bookmark=id.49x2ik5)*2:* Proportions of trials with the different target fixation patterns. Proportions depicted for (distractor-absent) trials on which the target appeared in the rare distractor region, separately for coincident (target at previous distractor position) and non-coincident target locations (target at previous non-distractor location), for the two participant groups (ASD vs. TD individuals)
